# Checklist of digeneans (Platyhelminthes, Trematoda, Digenea) of Georgia

**DOI:** 10.3897/BDJ.12.e110201

**Published:** 2024-01-08

**Authors:** Lela Arabuli, Lali Murvanidze, Anna Faltynkova, Levan Mumladze

**Affiliations:** 1 Institute of Zoology, Ilia State University, Tbilisi, Georgia Institute of Zoology, Ilia State University Tbilisi Georgia; 2 Mendel University in Brno, Brno, Czech Republic Mendel University in Brno Brno Czech Republic

**Keywords:** biodiversity, catalogue, Caucasus, helminths, host, parasite

## Abstract

**Background:**

In the present study, we aim to provide an inventory of digenetic trematodes (Platyhelminthes, Trematoda, Digenea) from Georgia including records from the freshwater, marine and terrestrial realms. The checklist is based on a critical review of data from 109 papers, 11 monographs and four Ph.D. theses published between 1935 and 2019 and our new records. The checklist includes information on synonymy, the host species, site of infection, geographical distribution and bibliographical references. The present data will serve as a baseline for further studies on trematodes from Georgia focused on integrative taxonomy, life-cycle elucidation, parasite ecology and epidemiology.

**New information:**

We compiled data on the digenean trematode fauna of Georgia, which is represented by 186 species (of these 173 identified to species level) belonging to 108 genera, 47 families and 17 superfamilies. This is the first checklist of the digeneans of Georgia. The majority of digenean species were recorded as adults (160 species), only a small fraction being found as cercariae (33 species) or metacercariae (24 species), in their first or second intermediate hosts, respectively. Predominantly, records of trematodes (62 species) from birds were found, followed by those parasitising fish (50 species, i.e. 32 species as adults and 18 as metacercariae), mammals (33 species) and amphibians (25 species, i.e. 23 species as adults and 2 as metacercariae), with the least number of species reported from reptiles (12 species, i.e. 9 species as adults and 3 as metacercariae). Adult digeneans recorded together with another life-cycle stage (metacercariae and/or cercariae) comprised 28 species, i.e. for 15% of the total trematode species number, a part of their life-cycle is known.

## Introduction

Digeneans (Platyhelminthes, Trematoda, Digenea) have complex life-cycles with several hosts involved (Cribb et al. 2003). They are parasites of all vertebrate groups and of a vast variety of invertebrates (Ditrich et al. 1997, Esteban et al. 1997, Dvořák et al. 1999, Faltýnková et al. 2009, Faltýnková et al. 2016, Manga-González and Ferreras 2019). Some groups of trematodes can be detrimental to human health and cause considerable loss to livestock (Ginetsinskaya 1968, Keiser and Utzinger 2009, Beesley et al. 2017, Esteban et al. 2019, Mas-Coma et al. 2019, Rizwan et al. 2022). However, trematodes are mainly important players in the functioning of ecosystems and they are an inherent part of food webs constituting a substantial part of biomass and, because of their sequencial use of intermediate and definitive hosts, they reflect the biodiversity of free-living host animals (Hechinger and Lafferty 2005).

Therefore, knowledge of trematode diversity and distribution, their life-cycles and transmission pathways is crucial not only for understanding the epidemiology and application of preventative measures, but also for biodiversity conservation (Dobson et al. 2008, Han et al. 2016, Selbach et al. 2020). Since Georgia is situated in the centre of the Caucasus biodiversity hotspot and in an area with a high degree of endemism for both vertebrate and invertebrate fauna (Myers et al. 2000, Mumladze et al. 2019), it is of vital importance to assess the species spectrum of the parasites which occur in this region.

In Georgia, the first studies on trematodes were conducted in the 1930s and early 1940s (Kamalov 1935, Burdjanadze 1937a, Burdjanadze 1937b, Chulkova 1939, Gamtsemlidze 1941, Kirshenblat 1941, Kurashvili 1941). Concerning digeneans of medical and veterinary importance, data on the distribution and prevalence of fascioliasis and dicrocoeliasis were published by Kurashvili and Rodonaia (1954). Since the 1950s, authors mainly focused their studies on the helminth fauna of specific host groups, i.e. fish (Kurashvili et al. 1951, Qoiava 1966, Petriashvili 1971, Kurashvili et al. 1973, Kurashvili et al. 1975, Kurashvili et al. 1990, Gogebashvili and Petriashvili 2002), amphibians and reptiles (Petriashvili 1964, Petriashvili 1966, Jankarashvili 1978, Jankarashvili 1985, Petriashvili et al. 1985), birds (Kurashvili 1953a, Kurashvili 1957, Kurashvili 1961b, Kurashvili et al. 1966, Kurashvili et al. 1976) and mammals (Rodonaia 1951, Matsaberidze 1961, Matsaberidze 1966a, Matsaberidze 1966b, Rodonaia 1966a, Rodonaia 1966b, Rodonaia 1971, Matsabaridze 1976). Fragmentary records of trematodes in freshwater and terrestrial molluscs serving as intermediate hosts were provided by several authors (Natsvlishvili 1968, Chiaberashvili and Javelidze 1968, Chiaberashvili 1971a, Chiaberashvili 1971b, Chiaberashvili and Javelidze 1977, Tkachenko 1988, Tkachenko 1990). The occurrence of human fascioliasis was examined by Gigitashvili (1965), Gigitashvili (1969), Zenaishvili et al. (2004a), Zenaishvili et al. (2004b) and Semyenova et al. (2006), who studied the genetic differentiation of European and Asian populations of *Fasciolahepatica* revealing that the Georgian population was of European origin and providing the only DNA sequences of trematodes from Georgia. In recent years, a few annotated checklists of helminths from different host taxa have been published by Murvanidze et al. (2008a), Murvanidze et al. (2008b) and Murvanidze et al. (2018).

Despite all the above-mentioned, a summarising review providing detailed information on the diversity of trematodes in Georgia has not been published so far. Only Kurashvili (1961a) reviewed the occurrence of some of the trematode species of medical and veterinary importance; and Kurashvili et al. (1980) published a monograph with a survey of parasites of fishes from the River Mtkvari (Kura), with digeneans included; however, there are only a few records which can be regarded as truly from the Georgian territory.

Thus, the aim of the present study was to compose a critically revised checklist of the digenetic trematodes recorded from Georgia. These data will serve as a baseline for future studies on biodiversity, taxonomy, life-cycles, ecological and epidemiological aspects of trematodes in Georgia.

## Materials and methods

For records of digeneans from Georgia, we searched the following databases: Google Scholar, Science Direct, Scopus and the Host-parasite database of the Natural History Museum London, using relevant keywords (‘(trematoda or digenea) and Georgia’; excluding records from Georgia, USA). Further, we searched for literary data in relevant papers and monographs. Data from monographs were checked with caution, because, in many cases, the information on geographical distribution was vague providing only general locations, for example, the rivers or mountain ranges expanding over more countries. The present checklist is based on 109 primary papers, 11 monographs and four Ph.D. theses published predominantly in Georgian or Russian language between 1935 and 2019 and our unpublished data.

We provide a parasite-host list with the parasites arranged in alphabetical order by superfamily and family; within each family, species are also listed alphabetically. The host species of each parasite are grouped by families and listed in alphabetical order by family and species. Each parasite taxon contains information on synonyms (listed under "Nomenclature" only when appearing in Georgian literature), host species, site of infection within hosts, geographical distribution and bibliographical references associated with the parasite and its hosts in Georgia. Global geographical distribution is mainly compiled from monographs of Skryabin (1947), Dubois (1968), Dubois (1970), Yamaguti (1971), Gibson et al. (2002), Niewiadomska (2003), Jones et al. (2005), Sitko et al. (2006), Bray et al. (2008) and Fauna Europea (de Jong et al. 2014), GBIF (GBIF.org 2022) and WoRMS (WoRMS 2022). The geographical localities, indicating the distribution of trematodes in Georgia, are listed in alphabetical order. Georgia is geographically divided by the Surami (Likhi) Range into two parts, Western Georgia (WG) and Eastern Georgia (EG); and the abbreviations (WG and EG) are used throughout the text to reflect this division. These regions have different climatic conditions – humid and mild climate in the Western and dry continental in the Eastern part of the country (Maruashvili 1964, Murvanidze and Mumladze 2016).

Life-cycle stages other than the sexual adults are indicated in parentheses following the host category, i.e. intramolluscan stages in first intermediate mollusc hosts comprising sporocysts, rediae and cercariae (the latter are asexual free-living stages emerging from the snail first intermediate hosts); and metacercariae encysted or unencysted in tissue of second intermediate or paratenic hosts.

Species names were checked for their current state and, for the nomenclature of all taxa, we refer to the Fauna Europea (de Jong et al. 2014), GBIF (GBIF.org 2022), WoRMS (WoRMS 2022), Catalogue of Life (Gibson et al. 2023) and Molluscabase (2023); for the classification of trematodes at family level, we follow the “Keys to the Trematoda” (Gibson et al. 2002, Jones et al. 2005, Bray et al. 2008) and, at the superfamily level, we follow WoRMS (2022) and Catalogue of Life (Gibson et al. 2023).

Trematodes not identified to species level were included in the checklist as well; in ambiguous cases, when it was impossible to discern the identity of the records (*Echinochasmus* sp., *Cephalogonimus* sp. and *Telorchis* sp.), they were listed with 'spp.'. However, we did not include taxa of doubtful identity, but we listed them in the Discussion with an explanation of their status and history; the names of these taxa are provided exactly as they occur in literature. Records of cercarial names, which are regarded as provisional, because they do not conform to the taxonomy of adult digeneans, were not included in the checklist either. However, to have a complete view on the potential biodiversity state of trematodes in Georgia, they are included in the Discussion provided with remarks on their occurrence.

Previously unpublished trematode specimens included in the checklist are deposited in the collections of the Institute of Zoology, Ilia State University.

## Checklists

### Annotated checklist of Digenea of Georgia

#### Phylum

Minot, 1876

91826A85-D4C8-5D33-991D-529D0B35DC5B

#### Trematoda

Rudolphi, 1808

84026605-FFA5-593E-A288-4F51737A1CAE

#### Digenea

Carus, 1863

C75D08BF-0208-5EE2-AE6E-F58F17FFEBFD

#### Diplostomida

Olson, Cribb, Tkach, Bray & Littlewood, 2003

627CA87A-4A31-5753-9083-85E94F7AC31A

#### Brachylaimoidea

Joyeux & Foley, 1930

092EAECF-778D-5205-B7A3-8FD4FFEDCA07

#### Brachylaimidae

Joyeux & Foley, 1930

45A2417A-6E40-5A57-BD5C-481AE9CCADE3

#### Brachylaima

Dujardin, 1843

829676F0-6467-520C-B1D9-AD87A41E117F

#### Brachylaima fulvum

Dujardin, 1843

BA636AD4-FED6-5CAA-B796-286581A9EE20

Panopistus europaeus Soltys, 1952

##### Parasite of

mammals - Soricidae: *Sorexaraneus*, *S.raddei*.

**Site of infection**: small intestine.

##### Distribution

Occurring in Europe; **in Georgia**: EG: Borjomi; WG: Batumi, Gagra, Oni, Tskaltubo reported by Matsaberidze (1966a), Matsaberidze (1966b), Matsaberidze (1967), Rodonaia (1971), Matsabaridze (1976) and Kurashvili (1984b).

#### Brachylaima fuscata

(Rudolphi, 1819) Joyeux, Baer & Timon-David, 1932

78595968-F303-5514-91D1-F6CD30E34855

Brachylaemus fuscatus (Rudolphi, 1819)

##### Parasite of

birds - Anatidae: *Mergellusalbellus*; Columbidae: *Columbalivia*; Phasianidae: *Meleagrisgallopavo*, *Phasianuscolchicuscolchicus*; Turdidae: *Turdusmerula*.

**Site of infection**: caecum, oesophagus, small intestine.

##### Distribution

Occurring in the Holarctic Region; **in Georgia**: EG: Marneuli, Martkhopi; WG: Samtredia reported by Kurashvili (1957), Kurashvili (1961a), Kurashvili et al. (1966), Matsaberidze (1966b), Japaridze and Savateeva (1967), Kurashvili et al. (1976), Kurashvili et al. (1977) and Kurashvili (1984b).

#### Brachylaima recurva

(Dujardin, 1845) Joyeux & Foley, 1930

3D2BE2FC-95E0-57F2-B9A9-4AC494CF49CF

##### Parasite of

mammals - Erinaceidae: *Erinaceuseuropaeus*; Gliridae: *Dryomysnitedula*; Muridae: *Apodemussylvaticus*.

**Site of infection**: small intestine.

##### Distribution

Occurring in Europe, Asia; **in Georgia**: EG: Borjomi, Khashuri, Tbilisi; WG: Abasha, Ambrolauri, Aphkhazeti, Kharagauli, Samtredia, Tskaltubo reported by Kirshenblat (1948), Matsaberidze (1966a), Matsaberidze (1966b), Rodonaia (1966a), Matsaberidze (1967), Matsabaridze (1976) and Kurashvili (1984b).

#### Brachylaima sp.

90848F3D-FA87-5C58-92BE-85B398FDD562

Brachylaemus sp. of Sharpilo (1962)

##### Parasite of

reptiles - Lacertidae: *Lacertastrigata*.

**Site of infection**: intestine.

##### Distribution

Cosmopolitan distribution; **in Georgia**: EG: Khashuri reported by Sharpilo (1962) and Murvanidze et al. (2008b).

#### fam. Brachylaimidae gen. sp.

F8FDE4A6-B631-561A-A139-AE3289EBE1FA

##### Parasite of

molluscs (intramolluscan stage) - Helicidae: *Helixlucorum*.

**Site of infection**: hepatopancreas.

##### Distribution

Cosmopolitan distribution; **in Georgia**: EG: Qvemo Qartli, Tbilisi; WG: Adjara, Imereti reported by Murvanidze et al. (2010) and Arabuli et al. (2019)

#### Ityogonimus

Lühe, 1899

3AA55595-BFA4-5300-994E-0862AA30182D

#### Ityogonimus talpae

(Goeze, 1782) Witenberg, 1925

6DBFD195-70E3-5E9D-81F6-CC8865DACA09

##### Parasite of

mammals - Talpidae:*Talpaeuropea*, *T.caucasica*, *T.orientalis*.

**Site of infection**: small intestine, stomach.

##### Distribution

Occurring in Europe; **in Georgia**: EG: Borjomi; WG: Abasha, Qobuleti, Samtredia, Sokhumi reported by Matsaberidze (1966b), Rodonaia (1966a), Matsaberidze (1967), Rodonaia (1971), Matsabaridze (1976) and Kurashvili (1984b).

#### Postharmostomum

Witenberg, 1923

1F0D89FF-AA3E-50A1-B34F-43D293845A5F

#### Postharmostomum commutatum

(Diesing, 1858) Skrjabin, 1923

362DE1F8-3258-5754-8601-246FCE46F2F2

##### Parasite of

birds - Phasianidae: *Meleagrisgallopavo*, *Tetraogalluscaucasicus*.

**Site of infection**: caecum, small intestine.

##### Distribution

Cosmopolitan distribution; **in Georgia**: EG: Kazbegi; WG: Samtredia reported by Dinnik (1938), Gushanskaia (1952), Kurashvili (1961a), Japaridze and Savateeva (1967) and Kurashvili et al. (1976).

#### Postharmostomum ularicum

(Kurashvilli, 1956)

47BAE5EC-4B24-5A31-A8EA-89C88BCF8438

##### Parasite of

birds - Phasianidae:*Tetraogalluscaucasicus*.

**Site of infection**: intestine.

##### Distribution

Recorded only in Georgia, in Yamaguti (1971) listed as in Russia; **in Georgia**: EG: Lagodekhi, Mtatusheti, Tianeti reported by Kurashvili (1956), Kurashvili (1957) and Khrustalev and Moskvin (2021).

#### Panopistidae

Yamaguti, 1958

C604B8F4-3746-5E62-9604-20EA568E923C

#### Pseudoleucochloridium

Pojmanska, 1959

AED359A0-2D1E-5C00-BEF9-B62F943EDB0B

#### Pseudoleucochloridium soricis

(Soltys, 1952) Pojmanska, 1959

00534CBB-02A9-5E6A-9D30-D97E0E1D5F7D

Leucochloridium soricis Soltys, 1952

##### Parasite of

mammals - Soricidae: *Sorexaraneus*, *S.minutus*, *S.raddei*.

**Site of infection**: small intestine.

##### Distribution

Occurring in Europe and Asia; **in Georgia**: EG: Borjomi; WG: Samtredia, Sokhumi, Qutaisi reported by Matsaberidze (1967), Rodonaia (1971), Matsabaridze (1976) and Kurashvili (1984b).

#### Diplostomoidea

Poirier, 1886

234F92DF-371D-535F-BE0B-C150ECA36175

#### Cyathocotylidae

Mühling, 1898

9FC6FD7C-22AE-5D8A-AB1A-62714F6E6428

#### Mesostephanus

Lutz, 1933

290881B6-2812-5112-B540-CE658DA67DF5

#### Mesostephanus appendiculatus

(Ciurea, 1916) Lutz, 1935

155EB04C-5A2B-56FB-8E50-718F14EECECB

##### Parasite of

mammals - Felidae: *Felischaus*.

molluscs (intramolluscan stage) - Melanopsidae: *Melanopsispraemorsa*.

**Site of infection**: intestine.

##### Distribution

Occurring in the Holarctic Region; **in Georgia**: EG: Gardabani; WG: Lanchkhuti, Rivers: Pichori, Rioni reported by Rodonaia (1971), Galaktionov et al. (1980) and Ataev and Dobrovolskiy (1992).

#### Paracoenogonimus

Katsurada, 1914

E1400A73-3897-561B-B190-DB5068A8DCFA

#### Paracoenogonimus ovatus

Katsurada, 1914

4889B573-3208-5746-B93F-7C9903356FB5

##### Parasite of

fishes (metacercariae) - Clupeidae: *Alosatanaica*, *A.immaculata*; Cyprinidae: *Abramisbrama*, *Barbuscapito*, *Cyprinuscarpio*, *Rutilusrutilus*, *Scardiniuserythrophthalmus*, *Vimbavimba*; Esocidae: *Esoxlucius*; Percidae: *Percafluviatilis*, *Sanderlucioperca*.

**Site of infection**: musculature, fins.

##### Distribution

Occurring in Europe; **in Georgia**: WG: Lakes: Didi Narionali, Paliastomi reported by Chernova (1973) and Murvanidze et al. (2018).

#### Szidatia

Dubois, 1938

A14462E3-0775-5DEB-8AC8-4B6079ED4AB1

#### Szidatia joyeuxi

(Hughes, 1929) Dubois, 1938

3A71A9D1-B679-5F00-9E4F-494760567DD8

##### Parasite of

reptiles - Colubridae: *Natrixnatrix*.

**Site of infection**: intestine.

##### Distribution

Occurring in Africa; **in Georgia**: WG: Khobi, Zugdidi – Anaklia reported by Jankarashvili (1985), Jankarashvili and Sharpilo (1985) and Murvanidze et al. (2018).

#### Diplostomidae

Poirier, 1886

27D0D566-F276-5534-BCB6-8D3560D8C339

#### Alaria

Schrank, 1788

4170CD5E-3B90-51BB-A6F9-0D5908157EA6

#### Alaria alata

(Goeze, 1782) Krause,1914

2BBDA9DD-5318-52CE-91F1-910FF09A6065

##### Parasite of

mammals - Canidae: *Canisaureus*, *C.lupus*, *C.lupusfamiliaris*, *Vulpesvulpes*; Mustelidae: *Martesfoina*.

reptiles (metacercariae) - Colubridae: *Natrixtessellata*.

**Site of infection**: intestine.

##### Distribution

Occurring in Eurasia, North and South America, Australia; **in Georgia**: EG: Adigeni, Akhalqalaqi, Borjomi, Dedoflistskaro, Gardabani, Marneuli, Sagarejo, Signagi, surroundings of Tbilisi, Tetritskaro. WG: Gali, Mtskheta, Zugdidi reported by Kamalov (1935), Gamtsemlidze (1941), Burjanadze (1943), Rodonaia (1951), Sharpilo (1962), Rodonaia (1966a), Rodonaia (1966b), Rodonaia (1971), Kurashvili (1984b) and Murvanidze et al. (2008b).

#### Bolbophorus

Dubois, 1935

5C79DBC9-FD34-5F2C-A114-AD03CD770EEF

#### Bolbophorus confusus

(Krause, 1914) Dubois, 1935

BD7AB656-B2DD-5FDC-83B6-2517FA43CFAC

##### Parasite of

fishes (metacercariae) - Cyprinidae: *Barbusbarbus*, *Squaliuscephalus*.

**Site of infection**: muscle.

##### Distribution

Occurring in the Holarctic, Ethiopian Regions, India, Australia; **in Georgia**: River Mtkvari reported by Kurashvili et al. (1980).

#### Codonocephalus

Diesing, 1850

D06FB98D-230B-5177-A993-C8961454312F

#### Codonocephalus urniger

(Rudolphi, 1819) Lühe, 1909

47707C57-8C64-5E0A-BC38-7E000487268A

##### Parasite of

amphibians (metacercariae) - Ranidae: *Pelophylaxridibundus*.

**Site of infection**: kidneys, mouth cavity, muscle.

##### Distribution

Palaearctic distribution; **in Georgia**: EG: Tbilisi – Samgori; WG: Bebesiri Lake, Ozurgeti, Samtredia reported by Chiaberashvili and Mchedlidze (1961), Petriashvili et al. (1985) and Murvanidze et al. (2008a).

#### Diplostomum

von Nordmann, 1832

12270BEB-5072-5C92-BFF6-1DD235EF00FE

#### Diplostomum commutatum

(Diesing, 1850) Dubois, 1937

0FEEA9A5-1598-5386-A898-83D262A84163

##### Parasite of

fishes (metacercariae) - Cyprinidae: *Rutilusrutilus*.

**Site of infection**: no data available.

##### Distribution

Holarctic distribution; **in Georgia**: WG: Narionali Lake reported by Chernova (1973) and Murvanidze et al. (2018).

#### Diplostomum spathaceum

(Rudolphi, 1819) Olsson, 1876

EEDDAA0D-53CD-59CC-A239-2AC9E9113E3C

##### Parasite of

birds - Laridae: *Laruscanus*, *L.minutus*, *Chroicocephalusridibundus*.

fishes (metacercariae) - Cyprinidae: *Abramisbrama*, *Acanthobramamicrolepis*, *Alburnusalburnus*, *A.fasciatus*, *A.hohenackeri*, *Ballerussapa*, *Barbusbarbus*, *Capoetasevangi*, *Carassiuscarassius*, *Chondrostomacyri*, *Ctenopharyngodonidella*, *Cyprinuscarpio*, *Gobiogobio*, *Leuciscusleuciscus*, *Luciobarbuscapito*, *L.mursa*, *Rutilusrutilus*, *R.rutiluskurensis*, *Scardiniuserythrophthalmus*, *Squaliuscephalus*, *Tincatinca*, *Varicorhinus* sp., *Vimbavimba*; Esocidae: *Esoxlucius*; Gobiidae: *Ponticolaconstructor*; Percidae: *Sanderlucioperca*.

molluscs (intramolluscan stage) - Lymnaeidae: *Lymnaeastagnalis*; *Peregrianaperegra*.

**Site of infection**: eye (fishes), small intestine (birds).

##### Distribution

With Holarctic distribution; **in Georgia**: EG: Rivers: Alazani, Aragvi basin, Mtkvari (Borjomi Gorge); Lakes: Bazaleti, Jandari, Kumisi, Khrami; Samgori, Tbilisi Reservoir; WG: Batumi, Madatapa Lake, River Mtkvari, Sokhumi reported by Kurashvili (1953a), Kurashvili (1957), Kurashvili (1961b), Chiaberashvili (1962a), Qoiava (1966), Chiaberashvili (1968), Petriashvili (1971), Kurashvili et al. (1973), Burtikashvili et al. (1978), Kurashvili et al. (1980), Kurashvili (1984a), Kurashvili (1984b), Kurashvili (1988), Nikolaishvili et al. (1990), Kurashvili et al. (1991), Gogebashvili and Petriashvili (2002), Arabuli et al. (2015), Japoshvili et al. (2017) and Murvanidze et al. (2018).

#### Diplostomum sp.

AC65045E-9848-5A17-BEAC-37C26B782F90

##### Parasite of

fishes (metacercariae) - Cyprinidae: *Abramisbrama*, *Alburnusalburnus*, *Capoetacapoeta*, *C.sevangi*, *Cyprinuscarpio*, *Rutilusrutiluskurensis*, *Scardiniuserythrophthalmus*, *Tincatinca*, *Vimbavimba*; Esocidae: *Esoxlucius*; Percidae: *Percafluviatilis*.

**Site of infection**: eye.

##### Distribution

Cosmopolitan distribution; **in Georgia**: River Mtkvari reported by Kurashvili et al. (1980).

#### Neodiplostomum

Railliet, 1919

FC0A9C64-F211-5A0D-887E-EB4C44377790

#### Neodiplostomum minor

(Dubois, 1936) Pearson, 1961

FABE07AA-85D7-5299-BE99-790EDF17EC38

##### Parasite of

reptiles (metacercariae) - Colubridae: *Natrixnatrix*, *N.tessellata*.

**Site of infection**: viscera.

##### Distribution

Cosmopolitan distribution; **in Georgia**: EG: Bazaleti Lake, Jinvali, Kazbegi, surroundings of Tbilisi; WG: Khobi, Zugdidi, surroundings of Batumi reported by Petriashvili (1966) and Jankarashvili (1985).

#### Posthodiplostomum

Dubois, 1936

A47542F7-8551-5E5D-8128-1E12056305C0

#### Posthodiplostomum brevicaudatum

(von Nordmann, 1832) Wisniewski, 1958

0AC79135-2498-59B2-B875-0750F051E812

Neascus brevicaudatus (von Nordmann, 1832) Hughes, 1928

##### Parasite of

fishes (metacercariae) - Esocidae: *Esoxlucius*.

**Site of infection**: musculature, skin.

##### Distribution

With Holarctic distribution; **in Georgia**: EG: Bazaleti Lake, Rivers: Alazani, Iori, Mtkvari; WG: Paliastomi Lake reported by Chiaberashvili (1968), Chernova (1973) and Murvanidze et al. (2018).

#### Posthodiplostomum cuticola

(von Nordmann, 1832) Dubois, 1936

FDD8FBA8-542B-5F57-A052-CC4BBA69A98B

##### Parasite of

fishes (metacercariae) - Cyprinidae: *Abramisbrama*, *Alburnuschalcoides*, *Ballerussapa*, *Chondrostomacyri*, *Cyprinuscarpio*, *Leuciscusaspius*, *Luciobarbuscapito*, *Scardiniuserythrophthalmus*, *Tincatinca*, *Vimbavimba*.

**Site of infection**: fins, gills, skin.

##### Distribution

Occurring in Europe and Asia; **in Georgia**: EG: River Mtkvari, Sagarejo reported by Kurashvili and Petriashvili (1977), Kurashvili et al. (1980), Kurashvili et al. (1983a), Kurashvili et al. (1990), Gogebashvili and Petriashvili (2002) and Murvanidze et al. (2018).

#### Posthodiplostomum sp.

1E381661-B806-5FB6-9708-84EFDCC93D8C

##### Parasite of

birds - Charadriidae: *Charadriusalexandrinus*.

**Site of infection**: intestine.

##### Distribution

Cosmopolitan distribution; **in Georgia**: EG: Bazaleti Lake reported by Kurashvili (1950) and Kurashvili (1957).

#### Tylodelphys

Diesing, 1850

5AA5B4EB-5A33-57EB-B56D-B6399548FA82

#### Tylodelphys clavata

(von Nordmann, 1832) Diesing, 1850

8F75C075-2A8A-5322-AF26-7A06B170CF25

Diplostomum clavatum Nordmann, 1832

##### Parasite of

fishes (metacercariae) - Cyprinidae: *Abramisbrama*, *Alburnoideseichwaldi*, *Alburnusalburnus*, *A.chalcoides*, *A.derjugini*, *Ballerussapa*, *Barbustauricusrionica*, *Bliccabjoerkna*, *Capoetacapoeta*, *C.sevangi*, *Chondrostomacolchicum*, *Cyprinuscarpio*, *Leuciscusleuciscus*, *L.cephalusorientalis*, *Luciobarbuscapito*, *Rhodeussericeus*, *Rutilusrutilus*, *Scardiniuserythrophthalmus*, *Tincatinca*, *Varicorhinus* sp., *Vimbavimba*; Esocidae: *Esoxlucius*; Percidae: *Percafluviatilis*, *Sanderlucioperca*.

**Site of infection**: eye.

##### Distribution

Holarctic distribution; **in Georgia**: EG: Rivers: Alazani, Aragvi Basin, Iori, Mtkvari; Lakes: Bazaleti, Jandari; Khrami Reservoir; WG: Rivers: Bebesiri, Kaparchina, Rioni, Shavi; Lakes: Japana, Paliastomi reported by Chiaberashvili (1955), Chiaberashvili (1957), Chiaberashvili (1959), Chiaberashvili (1962b), Qoiava (1966), Chiaberashvili (1968), Petriashvili (1971), Chernova (1973), Burtikashvili et al. (1978) and Murvanidze et al. (2018).

#### Tylodelphys sp.

B8B1A27B-A128-5499-9238-14B86BAE0E2C

##### Parasite of

molluscs (intramolluscan stage) - Lymnaeidae: *Radixeuphratica*.

**Site of infection**: body cavity, hepatopancreas.

##### Distribution

Cosmopolitan distribution; **in Georgia**: EG: Lisi Lake reported by Arabuli, unpublished data 2021.

#### Strigeidae

Railliet, 1919

5768B862-8C96-58DE-886A-6B52EFE0307B

#### Apatemon

Szidat, 1928

0759A688-DE37-51CE-9248-155A38BC54B5

#### Apatemon gracilis

(Rudolphi, 1819) Szidat, 1928

6A6A7583-A941-5C93-AFD5-A0D45C08F6EC

##### Parasite of

birds - Anatidae: *Anasplathyrynchos*, A.platyrhynchosf.domestica, *Anseralbifrons*, A.anserf.domestica, *Aythyaferina*, *Bucephalaclangula*, *Melanittafusca*, *Mergellusalbellus*.

**Site of infection**: small intestine.

##### Distribution

Holarctic distribution; **in Georgia**: EG: Dusheti – Bazaleti Lake, Gardabani – surroundings of Tbilisi Reservoir, Jandari (Karaiazi) Lake, Marneuli, Tsalka – Khrami Reservoir; WG: Abasha, Chkhorotsku, Khobi, Lanchkhuti – Mamati, Martvili, Poti, Tsalenjikha, Zugdidi reported by Kurashvili (1953a), Kurashvili (1957), Japaridze and Savateeva (1967), Kurashvili et al. (1976) and Kurashvili (1984b).

#### Apharyngostrigea

Ciurea, 1927

9B0DE9B5-F81B-59FB-8A78-8A942E95ACD9

#### Apharyngostrigea cornu

(Zeder, 1800) Ciurea, 1927

2E989642-0730-56AE-A87D-7429869717D5

##### Parasite of

birds - Ardeidae: *Egrettagarzetta*, *Nycticoraxnycticorax*; Scolopacidae: *Tringaochropus*.

fishes (metacercariae) - Cyprinidae: *Rutilusrutilus*, *Scardiniuserythrophthalmus*, *Tincatinca*.

**Site of infection**: abdominal cavity, muscle (fishes); small intestine (birds).

##### Distribution

Occurring in the Holarctic, India, Madagascar; **in Georgia**: EG: Dusheti – Bazaleti Lake, Gardabani – Jandari Lake, River Mtkvari; WG: Poti – Chaladidi, River Mtkvari reported by Kurashvili (1953a), Kurashvili (1957), Kurashvili et al. (1980) and Kurashvili (1984b).

#### Apharyngostrigea garciai

Tubangui, 1933

75065DEF-7AF1-53F8-95B3-8E3921CE2DB5

##### Parasite of

birds - Ardeidae: *Egrettagarzetta*.

**Site of infection**: small intestine.

##### Distribution

Occurring in Southeast Asia; **in Georgia**: EG: Dusheti – Bazaleti Lake, Gardabani – Jandari Lake reported by Kurashvili (1953a) and Kurashvili (1957).

#### Cotylurus

Szidat, 1928

F5692D1B-0452-532E-BB77-BB28A5A02D41

#### Cotylurus cornutus

(Rudolphi, 1809) Szidat, 1928

08A30D05-5D25-575C-B2A5-53DB46B67CF7

##### Parasite of

birds - Anatidae: *Anseralbifrons*, A.anserf.domestica, Anasplatyrhynchosf.domestica, *Aythyaferina*, *A.marila*, *Mergusmerganser*; Columbidae: *Columbalivia*; Charadriidae: *Vanelusvanellus*.

**Site of infection**: intestine, small intestine.

##### Distribution

Cosmopolitan distribution; **in Georgia**: EG: Dedoplistskaro – Eldari, Dusheti – Bazaleti Lake, Gardabani, Kumisi Lake, Marneuli, Qareli – Zguderi, Tianeti; WG: Abasha, Lanchkhuti – Mamati, Khobi, Lentekhi, Martvili, Paliastomi Lake, Poti, Samtredia, Tsalenjikha, Zugdidi – Axali kakhati reported by Kurashvili (1953a), Kurashvili (1957), Japaridze and Savateeva (1967), Kurashvili et al. (1973), Kurashvili et al. (1976) and Kurashvili (1984b).

#### Cotylurus hebraicus

Dubois, 1934

3238A63D-5948-5DFB-8454-5183A0DE5A2C

##### Parasite of

birds - Anatidae: Anasplathyrynchosf.domestica.

**Site of infection**: intestine.

##### Distribution

Occurring in the Holarctic Region and Brazil; **in Georgia**: WG: Samtredia – Ilori, Tsalenjikha reported by Japaridze and Savateeva (1967) and Kurashvili et al. (1976).

#### Ophiosoma

Szidat, 1928

7992B3F6-0E5E-58D2-9CB3-658DAB8E2900

#### Ophiosoma patagiatum

(Creplin, 1846) Dubois, 1937

07C909BB-08E8-5D89-82B5-7D60AECD8953

##### Parasite of

birds - Ardeidae: *Botaurusstellaris*; Laridae: *Chroicocephalusridibundus*.

**Site of infection**: intestine, small intestine.

##### Distribution

Occurring in Europe, Asia, Africa; **in Georgia**: EG: Kumisi Lake, Mtskheta – Natakhtari reported by Kurashvili (1953a), Kurashvili (1957) and Kurashvili et al. (1973).

#### Strigea

Abildgaard, 1790

F0ECB295-1A60-5B14-8133-15DDE1C16055

#### Strigea falconis

Szidat, 1928

153C139D-6761-5560-95A5-5417AD2CF21E

##### Parasite of

birds - Accipitridae: *Accipitergentiliscaucasicus*, *Aquilaclanga*, *Circus*; Anatidae: Anseranserf.domestica; Phasianidae: Gallusgallusf.domestica, *Meleagrisgallopavo*.

birds (metacercariae) - Anatidae: Anasplatyrhynchosf.domestica, Anseranserf.domesticus, Phasianidae: Gallusgallusf.domesticus, *Meleagrisgallopavo*.

**Site of infection**: intestine (adults); subcutaneous tissue of chest, neck and thighs (metacercariae).

##### Distribution

Holarctic Region, North Africa; **in Georgia**: EG: Borjomi, Gardabani, Kumisi Lake; WG: Gudauta reported by Kurashvili (1957), Japaridze and Savateeva (1967), Kurashvili et al. (1973), Kurashvili et al. (1976) and Kurashvili (1984b).

#### Strigea strigis

(Schrank, 1788) Abildgaard, 1790

4E5BD089-4EEE-5ED0-BBE5-5A2F7A97A10D

##### Parasite of

birds - Accipitridae: *Buteobuteo*, *Circusaeruginosus*, *Milvus* sp.

reptiles (metacercariae) - Colubridae: *Natrixnatrix*, *N.tesselata*.

amphinbians: (metacercariae) - Ranidae: *Pelophylaxridibundus*.

**Site of infection**: intestine.

##### Distribution

Occurring in the Palaearctic Region, Africa; **in Georgia**: EG: Bazaleti Lake, Jinvali, Khazbegi, surroundings of Tbilisi; WG: Khobi, surroundings of Batumi, Zugdidi reported by Petriashvili (1966) and Jankarashvili (1985).

#### Schistosomatoidea

Stiles & Hassall, 1898

A4DD7E35-53C4-57B2-A1C4-7EAB510EE5CA

#### Clinostomidae

Lühe, 1901

AF7AA396-778B-5686-860F-88FFC9AB4535

#### Clinostomum

Leidy, 1856

D263868B-0E1B-570C-844D-7A650C74E1B8

#### Clinostomum complanatum

(Rudolphi, 1814) Braun, 1899

7874F5A7-D48F-52C4-B4AE-352C203D8949

##### Parasite of

birds - Ardeidae: *Ardeacinerea*, *Egrettaalba*.

fishes (metacercariae) - Cyprinidae: *Alburnuschalcoides*, *Barbuscyri*, *Leuciscusaspius*, *Rutilusrutilus*, *Scardiniuserythrophthalmus*, *Squaliuscephalus*, *Tincatinca*, *Vimbavimba*; Esocidae: *Esoxlucius*; Percidae: *Percafluviatilis*, *Sanderlucioperca*; Siluridae: *Silurusglanis*.

**Site of infection**: oesophagus, oral cavity, pharynx.

##### Distribution

Cosmopolitan distribution; **in Georgia**: EG: Bazaleti Lake, Gardabani – Jandari (Karaiazi) Lake, River Mtkvari; WG: Ozurgeti, Lakes: Bebesiri, Paliastomi; River Rioni reported by Kurashvili (1953a), Chiaberashvili (1955), Chiaberashvili (1957), Kurashvili (1957), Kurashvili (1961a), Chiaberashvili (1962b), Chernova (1977), Kurashvili and Petriashvili (1977), Kurashvili et al. (1980), Kurashvili (1984b) and Murvanidze et al. (2018).

#### Clinostomum piscidium

Southwell & Prashad, 1918

8197D81C-4624-52D8-AD1D-EAA23F8E09F8

##### Parasite of

fishes (metacercariae) - Clupeidae: *Alosaimmaculata*; Mugilidae: *Lizasaliens*, *L.aurata*; Percidae: *Percafluviatilis*.

**Site of infection**: oesophagus, oral cavity.

##### Distribution

Cosmopolitan distribution; **in Georgia**: WG: Batumi, Black Sea coast, Sokhumi, surroundings of Poti; Lakes: Japana, Paliastomi reported by Kurashvili and Tabidze (1947), Kurashvili et al. (1951), Kurashvili (1961a), Kurashvili and Petriashvili (1977) and Murvanidze et al. (2018).

#### Euclinostomum

Travassos, 1928

45613EF8-85CB-57EE-B182-67ED55A14F2B

#### Euclinostomum heterostomum

(Rudolphi, 1809) Travassos, 1928

37573EF1-0115-5D50-B0E2-F97D5C9E6F36

Euclinostomum skrjabini Kurashvili, 1948

##### Parasite of

birds - Ardeidae: *Ardeacinerea*, *A.purpurea*.

**Site of infection**: oesophagus.

##### Distribution

In the Holarctic Region, India, Southeast Asia, Africa; **in Georgia**: EG: Dusheti – Bazaleti Lake, Tbilisi – Digomi, Tsalka reported by Kurashvili (1948), Kurashvili (1950), Kurashvili (1953a), Kurashvili (1957) and Kurashvili (1961a).

#### Schistosomatidae

Stiles & Hassall, 1898

63EA02C5-2B8E-5FB1-983D-2D339372C439

#### Bilharziella

Looss, 1899

AEB60863-2795-5BF4-8497-08991A6CC3AC

#### Bilharziella polonica

(Kowalewski, 1895) Looss, 1899

9C0345E9-FE76-58A8-A3A6-B7A28D72F519

##### Parasite of

birds - Anatidae: *Anascrecca*, *A.penelope*, A.plathyrynchosf.domestica.

**Site of infection**: abdominal cavity, blood vessels of liver.

##### Distribution

Holarctic distribution; **in Georgia**: EG: Dusheti – Bazaleti Lake, Gardabani – Tbilisi Sea, Kumisi Lake, Tsalka; WG: Mestia – Lenjeri, Zugdidi reported by Kurashvili et al. (1976) and Kurashvili (1984b).

#### Dendritobilharzia

Skrjabin & Zakharov, 1920

A79B9549-6FEF-55F0-B493-EB38CC13882C

#### Dendritobilharzia pulverulenta

(Braun, 1901) Skrjabin, 1924

44390F00-D0A7-55A2-AFB8-51700DE47AB5

##### Parasite of

birds - Anatidae: *Anascrecca*, *A.penelope*, A.plathyrynchosf.domestica, Anseranserf.domestica.

**Site of infection**: blood vessels – large veins.

##### Distribution

Holarctic distribution; **in Georgia**: EG: Gardabani – Tbilisi Sea, Lagodekhi, Lisi Lake; WG: Surroundings of Poti reported by Kurashvili et al. (1976).

#### Plagiorchiida

La Rue, 1957

2EDE2A38-DC27-52F9-BC99-B7D9E0132785

#### Azygioidea

Lühe, 1909

FB980C7A-60ED-59D5-9593-99A2FDBC1EE4

#### Azygiidae

Lühe, 1909

82889096-4801-5760-BE68-4403E39732C9

#### Azygia

Looss, 1899

1446670F-6043-5279-9916-02601D2977C5

#### Azygia lucii

(Müller, 1776) Lühe, 1909

C0A65FA5-985C-5E35-A191-9579BD7C0EEC

##### Parasite of

fishes - Esocidae: *Esoxlucius*; Percidae: *Sanderlucioperca*; Salmonidae: *Salmotruttalabrax*; Siluridae: *Silurusglanis*.

**Site of infection**: esophagus.

##### Distribution

Occurring in the Holarctic; **in Georgia**: WG: Japana Lake, Poti reported by Kurashvili and Tabidze (1947), Chernova (1973) and Murvanidze et al. (2018).

#### Bucephaloidea

Poche, 1907

18AB59AE-77F3-56F9-80E9-89AD0888CA61

#### Bucephalidae

Poche, 1907

6CCC7776-5941-5357-B141-B8306A60EEE3

#### Bucephalus

von Baer, 1827

97325F89-ABF8-5165-A0B7-82EC3A6FAFA0

#### Bucephalus polymorphus

von Baer, 1827

54BCE3B7-DAF8-591D-A31E-0973D2D65BFB

##### Parasite of

fishes - Cyprinidae: *Abramisbrama*, *Alburnuschalcoides*, *A.derjugini*, *Ballerussapa*, *Phoxinuscolchicus*, *Rutilusrutilus*, *Scardiniuserythrophthalmus*; Esocidae: *Esoxlucius*; Percidae: *Percafluviatilis*, *Sanderlucioperca*; Siluridae: *Silurusglanis*.

**Site of infection**: fins, gills, intestine.

##### Distribution

Occurring in the Holarctic Region; **in Georgia**: EG: River Mtkvari; WG: Rivers: Rioni, Shavi, rRver Shavi’s Fish-Factory; Bebesiri Lake reported by Chiaberashvili (1955), Chiaberashvili (1957), Chiaberashvili (1959), Chiaberashvili (1962b), Kurashvili et al. (1980) and Murvanidze et al. (2018).

#### Rhipidocotyle

Diesing, 1858

536126E7-8FD4-5425-BF82-4635DAB25EFD

#### Rhipidocotyle campanula

(Dujardin, 1845) Dollfus, 1968

121110CF-E07A-59D0-B1D3-EA2A90878E92

Rhipidocotyle illense (Ziegler, 1883) Dyk, 1954

##### Parasite of

fishes - Cyprinidae: *Abramisbrama*, *Bliccabjoerkna*, *Rhodeus*; Esocidae: *Esoxlucius*; Gobiidae: *Gobius*; Percidae: *Sanderlucioperca*; Siluridae: *Silurusglanis*.

**Site of infection**: fins, gills, intestine.

##### Distribution

Occurring in Europe, Asia; **in Georgia**: River Mtkvari; WG: Lakes: Didi Narionali, Paliastomi reported by Chernova (1973), Kurashvili et al. (1980) and Murvanidze et al. (2018).

#### Echinostomatoidea

Looss, 1902

44F64DFC-D1DF-5E00-AA6B-C35092502D65

#### Cyclocoelidae

Stossich, 1903

D1FA0CD8-F518-53FF-9121-A85C8941F82D

#### Harrahium

Witenberg, 1926

C0636594-CE4C-5062-9598-04B348BB1C20

#### Harrahium halli

(Harrah, 1922) Witenberg, 1926

31BFFBB1-FA8A-578F-A5F9-B973B8363328

Cyclocoelum halli Harrah, 1922

##### Parasite of

birds - Anatidae: *Anasquerquedula*.

**Site of infection**: abdominal cavity, air sacs of lungs.

##### Distribution

Occurring in North America; **in Georgia**: EG: Mtkvari River, Mtskheta reported by Kurashvili (1957) and Kurashvili (1961a).

#### Hyptiasmus

Kossack, 1911

7C242257-7DBF-5827-80C7-BC2414B54E60

#### Hyptiasmus magniproles

Witenberg, 1928

0E1A80B5-AFFF-556E-976C-BA1609DD0AEB

##### Parasite of

birds - Anatidae: *Mergellusalbellus*.

**Site of infection**: infraorbital cavity, nasal cavity.

##### Distribution

Occurring in Asia; **in Georgia**: EG: Marneuli reported by Kurashvili (1957).

#### Morishitium

Witenberg, 1928

B4FC8D89-DF56-5F26-ADE0-8491DCB52076

#### Morishitium bivesiculatum

(Prudhoe, 1944) Yamaguti, 1958

02EA1BA3-7B99-5198-BB7E-A309B6B7A03F

Cyclocoelum (Pseudhyptiasmus) bivesiculatum Prudhoe, 1944

##### Parasite of

birds - Turdidae: *Turdusmerulaaterrimus*.

**Site of infection**: abdominal cavity.

##### Distribution

Occurring in Asia; **in Georgia**: EG: Lagodekhi National Park reported by Kurashvili (1957) and Kurashvili (1961a).

#### Selfcoelum

Dronen, Gardner & Jiménez, 2006

1EFB2145-6FC5-506A-AE0C-9C3E1494D136

#### Selfcoelum orientale

(Skrjabin, 1913) Dronen & Blend, 2015

7E9C4E69-08E4-5237-9465-0D0448AE3739

Cyclocoelum orientale Skrjabin, 1913

##### Parasite of

birds - Scolopacidae: *Tringaglareola*; Turdidae: *Turdusphilomelos*, *T.merulaaterrimus*.

**Site of infection**: abdominal cavity, air sacs of lungs.

##### Distribution

Recorded in Turkestan; **in Georgia**: EG: Borjomi reported by Bayer (1941), Kurashvili (1957) and Kurashvili (1961a).

#### Skrjabinocoelum

Kurashvili, 1953

B7E35695-37DE-5829-8D80-6FC08A63E0EF

#### Skrjabinocoelum petrowi

Kurashvili, 1953

E1C3B574-7857-5ACA-9993-663EF7F7D366

##### Parasite of

birds - Scolopacidae: *Lymnocryptesminimus*.

**Site of infection**: abdominal cavity, body cavity.

##### Distribution

Occurring in the Caucasus Region - Georgia and Azerbaijan; **in Georgia**: EG: Bolnisi reported by Kurashvili (1953a), Kurashvili (1953b), Kurashvili (1957) and Kurashvili (1961a).

#### Echinochasmidae

Odhner, 1910

DCE13A1B-1EA3-5F97-8F7F-FE892B07CB09

#### Echinochasmus

Dietz, 1909

2B8BC145-71F0-57C0-9F6A-376B1CF13589

#### Echinochasmus dietzevi

Issaitschikov, 1927

4934DDE8-F9C8-5BFF-A8AB-B9974852A403

##### Parasite of

birds - Anatidae: *Aythyanyroca*; Podicipedidae: *Colymbuscaspicus*, *Podicepscristatus*.

**Site of infection**: small intestine.

##### Distribution

Palaerctic distribution; **in Georgia**: EG: Samgori; WG: Batumi, Poti – Rioni Valley reported by Kurashvili (1957), Kurashvili (1961a), Kurashvili (1961b) and Kurashvili (1984b).

#### Echinochasmus mathevossianae

Schakhtakhtinskaya in Kurashvili, 1957

0E2BF30D-521E-5294-A738-7D807A021F2D

##### Parasite of

birds - Anatidae: *Aythyafuligula*, *Nettarufina*.

**Site of infection**: bursa Fabricii, intestine.

##### Distribution

Occurrence recorded only in Georgia; **in Georgia**: EG: Tsalka (Khrami) Reservoir; WG: Sokhumi reported by Kurashvili (1957), Kurashvili (1961a) and Kurashvili (1984b).

#### Echinochasmus perfoliatus

(Ratz, 1908) Gedoelst, 1911

3440950E-4BC9-5F45-B055-7AFBC0D4489F

##### Parasite of

mammals - Canidae: *Canislupusfamiliaris*.

fishes (metacercariae) - Cyprinidae: *Alburnusalburnus*, *Alburnusfilippii*.

**Site of infection**: gills, small intestine.

##### Distribution

Occurring in the Holarctic region; **in Georgia**: EG: Tbilisi; WG: Batumi reported by Burdjanadze (1937a), Gamtsemlidze (1941), Burjanadze (1943), Kurashvili (1961a), Kurashvili et al. (1980) and Kurashvili (1984b).

#### Echinochasmus spp.

01466291-D58B-5AE1-9753-AB5CA16DBA21

##### Parasite of

fishes (metacercariae) - Cyprinidae: *Luciobarbusescherichii*, *Squaliuscephalus*.

mollusc (intramolluscan stages) - Melanopsidae: *Melanopsispraemorsa*.

**Site of infection**: gills.

##### Distribution

Cosmopolitan distribution; **in Georgia**: WG: Rivers: Pichori, Rioni reported by Chiaberashvili (1962b), Olenev and Dobrovolsky (1975), Kurashvili (1984a) and Manafov (2011).

#### Stephanoprora

Odhner, 1902

AB4C80EA-029C-58B8-9D57-D3352E4C2C71

#### Stephanoprora pseudoechinata

(Olsson, 1876) Yamaguti, 1958

A6EFB897-5736-5F77-96B1-1C1AF7B44E75

Mesorchis pseudoechinatus (Olsson, 1876) Dietz, 1909

##### Parasite of

birds - Anatidae: *Aythyanyroca*; Laridae: *Hydrocoloeusminutus*, *Laruscanus*.

**Site of infection**: small intestine.

##### Distribution

Occurring in the Holarctic, Africa; **in Georgia**: EG: Kumisi Lake, Lagodekhi, Marneuli; WG: River Kaparchina, Rioni Valley – Poti, Sokhumi reported by Kurashvili (1950), Kurashvili (1953a), Kurashvili (1957), Kurashvili (1961a) and Kurashvili (1984b).

#### Echinostomatidae

Looss, 1899

DD1C3E7F-4573-5E0D-BC7F-F556D7D8D497

#### Chaunocephalus

Dietz, 1909

8DE7CC82-FFCE-5A07-A690-476C6DAA0415

#### Chaunocephalus ferox orientalis

Baschkirova, 1941

C540879B-E422-514D-A638-6334E6ABFF7E

##### Parasite of

birds - Ciconiidae: *Ciconiaciconia*, *Ciconia* sp.

**Site of infection**: small intestine.

##### Distribution

Occurring in Eurasia, Australia; **in Georgia**: EG: Sagarejo, surroundings of Tbilisi; WG: surroundings of Samtredia reported by Kurashvili (1941), Kurashvili (1957) and Kurashvili (1984b).

#### Echinoparyphium

Dietz, 1909

CBE849C0-7757-53D3-BB57-61DB4293C2BE

#### Echinoparyphium colchicum

Javelidze, 1958

E2ED98C0-3741-5615-8A3D-98093459BCA8

##### Parasite of

molluscs (intramolluscan stage) - Viviparidae: *Viviparusviviparus*.

**Site of infection**: reproductive organs and ducts.

##### Distribution

Recorded only in Georgia; **in Georgia**: WG: freshwaters reported by Javelidze (1958).

#### Echinoparyphium mordwilkoi

Skrjabin, 1915

9F90BA57-D1A3-5BFA-971F-26A2C5AE388C

##### Parasite of

birds - Scolopacidae: *Scolopaxrusticola*, *Tringaochropus*.

**Site of infection**: intestine.

##### Distribution

Occurring in Asia; **in Georgia**: WG: Poti – Chaladidi reported by Kurashvili (1957), Kurashvili (1961a), Kurashvili et al. (1976) and Kurashvili (1984b).

#### Echinoparyphium recurvatum

(von Linstow, 1873) Lühe, 1909

B3F95E80-807A-5909-AC72-0F2EFD14B4FA

##### Parasite of

birds - Anatidae: *Anasacuta*, *A.crecca*, *A.platyrhynchos*, A.platyrhynchosf.domestica, *Anseranser*, *Marmaronettaangustirostris*; Ardeidae: *Botaurusstellaris*; Phasianidae: Gallusgallusf.domestica.

molluscs (intramolluscan stage) - Planorbidae: *Ancylusfluviatilis*, *Planorbisplanorbis*.

**Site of infection**: caecum, small intestine.

##### Distribution

Cosmopolitan distribution; **in Georgia**: EG: Dusheti – Bazaleti Lake, Gardabani, Marneuli, Sartichala, Tsalka; WG: Abasha, Khobi, Lanchkhuti – Mamati, Poti – River Kaparchina, Samtredia, Senaki, Tsageri, Tsalenjikha reported by Kurashvili (1957), Kurashvili (1961a), Japaridze and Savateeva (1967), Chiaberashvili (1971a), Kurashvili et al. (1976) and Kurashvili (1984b).

#### Echinostoma

Rudolphi, 1809

7413F898-07D1-5F61-8C91-D9506F4A7A4B

#### Echinostoma miyagawai

Ishii, 1932

9A391A73-D8D6-5736-B207-8BF2B0A0AF21

Echinostoma robustum Yamaguti, 1935

##### Parasite of

birds - Anatidae: *Anasplatyrhynchos*, A.platyrhynchosf.domestica, Anseranserf.domestica, *Nettarufina*; Columbidae: *Spilopeliachinensis*, *Streptopeliaturtur*; Phasianidae: Gallusgallusf.domestica, *Meleagrisgallopavo*.

molluscs (intramolluscan stage) - Lymnaeidae: *Ampullaceanalagotis*.

**Site of infection**: hepatopancreas, intestine.

##### Distribution

Occurring in Europe, Asia; **in Georgia**: EG: Dedoflistskaro – Qvemo qedi, Dusheti – Bazaleti Lake, Gardabani – Jandari Lake, Marneuli, Mtskheta, Sartichala, Tbilisi – Samgori, Lisi Lake, Shuakhevi, Tetriwkaro – Koda, Tsalka; WG: Paliastomi Lake, Qareli, Rioni Valley, Samtredia, Sokhumi, Terjola, Zugdidi reported by Kurashvili (1941), Kurashvili (1953a), Chiaberashvili (1954), Chiaberashvili (1957), Kurashvili (1961a), Japaridze (1962), Japaridze and Savateeva (1967), Chiaberashvili (1971a), Kurashvili et al. (1976) and Kurashvili (1984b).

#### Echinostoma paraulum

Dietz, 1909

B18DF2FA-AF54-5D3E-A8F3-33B0D810482C

##### Parasite of

birds - Anatidae: *Anasclypeata*, *A.platyrhynchos*, A.platyrhynchosf.domestica, Anseranserf.domestica, *Aythyaferina*, *Cygnuscygnus*, *Nettarufina*; Columbidae: *Columbalivia*.

molluscs (intramolluscan stage) - Lymnaeidae: *Ampullaceanabalthica*.

**Site of infection**: hepatopancreas, intestine.

##### Distribution

Occurring in Europe, Asia; **in Georgia**: EG: Gardabani, Sartichala, Tskhinvali; WG: Lanchkhuti, lowland of Rioni, Samtredia, surroundings of Poti, Tetritskaro, Zugdidi reported by Kurashvili (1950), Kurashvili (1953a), Chiaberashvili (1954), Kurashvili (1957), Kurashvili (1961a), Japaridze and Savateeva (1967), Chiaberashvili (1971a), Kurashvili et al. (1976) and Kurashvili (1984b).

#### Echinostoma revolutum

(Fröhlich, 1802) Looss, 1899

4CF208B1-7448-510E-BD3C-07CF0728A9DD

##### Parasite of

birds - Anatidae: *Anasclypeata*, *A.platyrhynchos*, A.platyrhynchosf.domestica, Anseranserf.domestica, *Mergellusalbellus*, *Mergusmerganser*, *M.serrator*; Ardeidae: *Ardeacinerea*; Phasianidae: Gallusgallusf.domestica.

molluscs (intramolluscan stage) - Lymnaeidae: *Ampullaceanabalthica*, *A.lagotis*, *Peregrianaperegra*, *Galbatruncatula*; Planorbidae: *Planorbisplanorbis*.

**Site of infection**: intestine.

##### Distribution

Occurring in the Holarctic Region; **in Georgia**: EG: Bazaleti Lake, Sarthichala, Tbilisi – Samgori, River Iori; WG: Batumi, Lentekhi, Mestia, Paliastomi Lake, Samtredia reported by Kirshenblat (1941), Burjanadze (1943), Chiaberashvili (1954), Kurashvili (1957), Kurashvili (1961a), Japaridze (1962), Japaridze and Savateeva (1967), Chiaberashvili (1971a), Kurashvili et al. (1973), Kurashvili et al. (1983b) and Kurashvili (1984b).

#### Echinostoma stantschinskii

Semenov, 1927

C19D21FA-DBF9-5AD0-A9B1-E1B84D465D04

##### Parasite of

birds - Scolopacidae: *Gallinagogallinago*.

**Site of infection**: large intestine.

##### Distribution

Occurring in Asia; **in Georgia**: WG: Poti – valley of Rioni reported by Kurashvili (1953a), Kurashvili (1957), Kurashvili (1961a) and Kurashvili et al. (1976).

#### Hypoderaeum

Dietz, 1909

F315C58A-C6BC-5CD7-AF03-3F9F3E834B68

#### Hypoderaeum conoideum

(Bloch, 1782) Dietz, 1909

A7AA8920-CABB-5EA9-8830-9026450124FE

##### Parasite of

birds - Anatidae: *Anasacuta*, *A.platyrhynchos*, A.platyrhynchosf.domestica, *Anseranser*, A.anserf.domestica, *Mergusmerganser*; Phasianidae: Meleagrisgallopavof.domestica; Scolopacidae: *Scopolaxrusticola*, *Tringaochropus*.

**Site of infection**: small intestine.

##### Distribution

Holarctic distribution; **in Georgia**: EG: Dmanisi, Dusheti – Bazaleti Lake, Gardabani, Lisi Lake, Samgori; WG: Batumi, Khobi, Ozurgeti, Poti, Samtredia reported by Kurashvili (1941), Kurashvili (1957), Kurashvili (1961a), Japaridze and Savateeva (1967), Kurashvili et al. (1976) and Kurashvili (1984b).

#### Hypoderaeum gnedini

Baschkirova, 1941

F8469D7D-1BEC-5189-8742-30B6F11FE03F

##### Parasite of

birds - Anatidae: *Anasplatyrhynchos*, A.platyrhynchosf.domestica, *Nettarufina*; Podicipedidae: *Podicepscristatuscristatus*; Rallidae: *Fulicaatra*.

**Site of infection**: intestine.

##### Distribution

Occurring in Asia (Azerbaijan); **in Georgia**: EG: Lagodekhi – Alazani Valley; WG: Lachkhuti, Poti – Rioni Valley, Samtredia reported by Kurashvili (1957), Kurashvili (1961a), Japaridze and Savateeva (1967) and Kurashvili et al. (1976).

#### Hypoderaeum vigi

Baschkirova, 1941

6F64B605-FC17-5862-9B10-807A93096C14

##### Parasite of

birds - Anatidae: *Anasplatyrhynchos*, A.platyrhynchosf.domestica.

**Site of infection**: intestine.

##### Distribution

Occurring in Asia (Kazakhstan); **in Georgia**: WG: Samtredia reported by Japaridze and Savateeva 1967, Kurashvili et al. (1976) and Kurashvili (1984b).

#### Moliniella

Hübner, 1939

4D3AD219-29BA-5CE2-AB2F-5A60C63DB3A4

#### Moliniella anceps

(Molin, 1859) Hübner, 1939

DDD6B209-BD92-5875-BE3D-F85F85352633

##### Parasite of

molluscs (intramolluscan stage) - Lymnaeidae: *Lymnaeastagnalis*.

**Site of infection**: hepatopancreas.

##### Distribution

Occurring in Europe; **in Georgia**: WG: Madatapa Lake reported by Arabuli et al. (2015).

#### Neopetasiger

Baschkirova, 1941

1B8015AB-A2E5-5556-9F1F-E51385E301F8

#### Neopetasiger megacanthus

(Kotlán, 1922) Tkach, Kudlai & Kostadinova, 2016

3C198ACA-09B4-57AF-AB0F-BE89430E2622

Petasiger megacanthus (Kotlán, 1922) Pande, 1939

##### Parasite of

birds - Anatidae: *Aythyanyroca*; Podicipedidae: *Podicepscristatus*.

**Site of infection**: caecum, small intestine.

##### Distribution

Occurring in the Palaearctic; **in Georgia**: WG: Batumi, Gudauta, Poti, Rioni lowland, River Kaparchina reported by Kurashvili (1957), Kurashvili (1961a), Kurashvili (1961b) and Kurashvili (1984b).

#### Patagifer

Dietz, 1909

0E29FBD2-3157-5CA4-84E1-B81A96AA3B38

#### Patagifer bilobus

(Rudolphi, 1819) Dietz, 1909

CBF1A520-AB4B-5B96-9ED2-32A15B1486C6

##### Parasite of

birds - Threskiornithidae: *Plegadisfalcinellus*.

**Site of infection**: intestine, stomach.

##### Distribution

Cosmopolitan distribution; **in Georgia**: EG: Bazaleti Lake, Gardabani; WG: Paliastomi Lake reported by Kurashvili (1957), Kurashvili (1961a) and Kurashvili (1984b).

#### Patagifer sp.

D65D93CA-05B6-5D5A-B6AF-AC7659988587

##### Parasite of

birds - Threskiornithidae: *Platalealeucorodia*.

**Site of infection**: small intestine.

##### Distribution

Cosmopolitan distribution; **in Georgia: EG**: Lisi Lake reported by Kurashvili (1957).

#### Pegosomum

Ratz, 1903

3784F076-7FF9-530B-BC04-8FF1EE19E1A0

#### Pegosomum petrowi

Kurashvili, 1949

ADA49048-2C38-550F-8ADD-9825442949A3

##### Parasite of

birds - Ardeidae: *Egrettaalba*.

**Site of infection**: gallbladder.

##### Distribution

Records exist only from Georgia; **in Georgia**: EG: Kumisi Lake, Lagodekhi, Marneuli reported by Kurashvili (1949), Kurashvili (1950), Kurashvili (1953a), Kurashvili (1957) and Kurashvili (1961a).

#### Pegosomum skrjabini

Shakhtakhtinskaya, 1949

1CCC7959-809D-50A9-A723-72869312E206

##### Parasite of

birds - Ardeidae: *Ardeacinerea*, *Egrettaalba*.

**Site of infection**: gallbladder, liver.

##### Distribution

Occurring in Asia (Azerbaijan); **in Georgia**: EG: Gardabani, Marneuli reported by Kurashvili (1950), Kurashvili (1953a), Kurashvili (1957) and Kurashvili (1961a).

#### Petasiger

Dietz, 1909

C9C2EA5C-B0B6-523B-BF9B-00B88E06847B

#### Petasiger exaeretus

Dietz, 1909

E0C23EE6-B5C1-5059-9DB0-1EE539EA92BA

##### Parasite of

birds - Phalacrocoracidae: *Phalacrocoraxcarbo*.

**Site of infection**: small intestine.

##### Distribution

Eurasia, Africa, Australia; **in Georgia**: EG: Gardabani – Jandari (Karaiazi) Lake; WG: Surroundings of Sokhumi reported by Kurashvili (1941), Kurashvili (1957), Kurashvili (1961a) and Kurashvili (1984b).

#### Petasiger radiatus

(Dujardin, 1845) Tkach, Kudlai & Kostadinova, 2016

D10873E4-9B67-5C0D-8171-DDCE864DDB05

Paryphostomum radiatum (Dujardin, 1845) Dietz, 1909

##### Parasite of

birds - Phalacrocoracidae: *Phalacrocoraxcarbo*.

**Site of infection**: intestine.

##### Distribution

Cosmopolitan distribution; **in Georgia**: EG: Gardabani reported by Kurashvili (1941), Kurashvili (1957) and Kurashvili (1961a).

#### Fasciolidae

Railliet, 1895

3CE96C67-D38E-5D1A-9DBA-638846256DD3

#### Fasciola

Linnaeus, 1758

95587A5B-E962-5136-BC44-FA1405F820AB

#### Fasciola gigantica

Cobbold, 1855

509E966A-DE2D-58EC-BE72-1F9F25F9FB2C

##### Parasite of

mammals - Bovidae: *Bostaurus*, *Ovisaries*; Cervidae: *Capreoluscapreolus*.

**Site of infection**: hepatic duct, liver.

##### Distribution

Cosmopolitan distribution; **in Georgia**: EG: Dusheti, Tsalka; WG: Gudauta, Khobi, Lanchkhuti, Poti, Samtredia, Senaki, Zugdidi reported by Gamtsemlidze (1941), Burjanadze and Baratashvili (1941), Burjanadze (1943), Kurashvili and Rodonaia (1954), Kurashvili (1961a), Rodonaia (1962), Rodonaia (1966a), Rodonaia (1971) and Kurashvili (1984b).

#### Fasciola hepatica

Linnaeus, 1758

A078F2C2-BD9B-5F44-A59F-4640AD01E91F

##### Parasite of

mammals - Bovidae: *Bostaurus*, *Bubalusbubalis*, *Caprahircus*, *Ovisaries*; Cervidae: *Capreoluscapreolus*; Equidae: *Equuscaballus*; Leporidae: *Lepuseuropaeus*; Myocastoridae: *Myocastorcoypus*; Suidae: *Susscrofa*.

molluscs (intramolluscan stage) - Lymnaeidae: *Galbatruncatula*, *Lymnaeastagnalis*; Sphaeriidae: *Pisidium* sp.

**Site of infection**: gallbladder, hepatic ducts, hepatopancreas, intestine, liver, lungs.

##### Distribution

Cosmopolitan distribution; **in Georgia**: EG: Akhaltsikhe, Akhmeta, Bakuriani, Dusheti, Gardabani, Gori, Samgori, Signagi, Tbilisi, Telavi; WG: Chiatura, Batumi, Khobi, Khulo, Kobuleti, Kutaisi, Lanchkhuti, Poti, Qobuleti, River Shavtskali, Samtredia, Senaki, Sokhumi, Tsalenjikha, Vani, Zugdidi reported by Gamtsemlidze (1941), Burjanadze (1943), Kurashvili and Rodonaia (1954), Qoiava (1956a), Qoiava (1956b), Qoiava (1961), Kurashvili (1961a), Rodonaia (1962), Chiaberashvili (1964), Rodonaia (1966a), Rodonaia (1966b), Rodonaia (1971), Chiaberashvili (1971a), Kurashvili et al. (1983b), Kurashvili (1984a) and Kurashvili et al. (1990).

#### Philophthalmidae

Looss, 1900

57FA89E4-7C51-55DB-A352-5C6E80F97B9F

#### Philophthalmus

Looss, 1899

8C3C9AB5-D611-5A74-A75D-62D651712F9A

#### Philophthalmus rhionica

Tichomirov, 1976

6988D724-3ABF-5B7E-BFAE-D87C90BDE57A

##### Parasite of

molluscs (intramolluscan stage) - Melanopsidae: *Melanopsispraemorsa*.

**Site of infection**: hepatopancreas.

##### Distribution

Recorded in Georgia only; **in Georgia**: WG: River Rioni reported by Tichomirov (1976), Ataev (1991) and Ataev and Dobrovolskiy (1992).

#### Psilostomidae

Looss, 1900

4B7FEABC-57A3-5BC9-AAEC-F3CA0FC97538

#### Psilochasmus

Lühe, 1909

050A06AF-8D4D-5119-91EF-4D271318DAEA

#### Psilochasmus longicirratus

Skrjabin, 1913

5B345F7C-9525-5DF4-9BE3-67D6C054E9C5

##### Parasite of

birds - Anatidae: *Anasacuta*, *A.clypeata*, Anasplatyrhynchosf.domestica, Anseranserf.domestica, *Aythyanyroca*.

**Site of infection**: caecum, intestine.

##### Distribution

Europe, North America; **in Georgia**: EG: Dusheti – Bazaleti Lake, Tsalka – Khrami Reservoir. WG: Gurianta, Khobi, Ozurgeti – Gomi, Samtredia, surroundings of Poti reported by Kurashvili (1957), Kurashvili (1961a), Japaridze and Savateeva (1967), Kurashvili et al. (1976) and Kurashvili (1984b).

#### Psilochasmus oxyurus

(Creplin, 1825) Lühe, 1909

DAE74651-9706-5733-91EF-036F5E5D335A

##### Parasite of

birds - Anatidae: *Anasacuta*, *Anasplathyrynchos*, *Tadornatadorna*; Phasianidae: Gallusgallusf.domestica.

**Site of infection**: intestine.

##### Distribution

North America, Europe, Asia (Iraq); **in Georgia**: EG: Lagodekhi – Alazani Valley; WG: Khobi, Rioni Valley, Samtredia, Senaki, surroundings of Poti reported by Kurashvili (1953a), Kurashvili (1957), Kurashvili (1961a), Japaridze and Savateeva (1967), Kurashvili et al. (1976) and Kurashvili (1984b).

#### Typhlocoelidae

Harrah, 1922

F2DF9A95-2143-5BD0-89D0-7BB2A75FD6C6

#### Tracheophilus

Skrjabin, 1913

E1C3FF95-6EB6-5A68-BAD4-92542AAC0525

#### Tracheophilus sisowi

Skrjabin, 1913

220B9710-AFF8-5E9F-B379-0E30EA1DE9E2

##### Parasite of

birds - Anatidae: *Anasacuta*, *A.clypeata*, *A.platyrhynchos*, A.platyrhynchosf.domestica, *A.querquedula*, Anseranserf.domestica, *Aythyafuligula*.

**Site of infection**: abdominal cavity, lungs, trachea.

##### Distribution

Holarctic distribution; **in Georgia**: EG: Bolnisi, Dusheti – Bazaleti Lake, Gardabani, Marneuli, Tetritskaro. WG: Samtredia reported by Burjanadze (1943), Kurashvili (1957), Kurashvili (1961a), Japaridze and Savateeva (1967) and Kurashvili et al. (1976).

#### Gorgoderoidea

Looss, 1901

2B2E7F6A-693D-5046-9192-B2813D64C237

#### Allocreadiidae

Looss, 1902

12E62025-C4D8-5845-87BA-4A975FE0AFB9

#### Allocreadium

Looss, 1900

6D9B4A20-A00B-52EC-A704-EC0CD76A4C36

#### Allocreadium dogieli

Koval, 1950

5E5C9861-D03F-597C-9528-0AE75342B082

##### Parasite of

fishes - Cyprinidae: *Luciobarbusmursa*.

**Site of infection**: intestine.

##### Distribution

Occurring in Europe; **in Georgia**: EG: River Alazani reported by Chiaberashvili (1968) and Murvanidze et al. (2018).

#### Allocreadium isoporum

(Looss, 1894) Looss, 1902

C6A355A7-44F9-58D6-AFAE-F336B7FFC250

##### Parasite of

fishes - Cyprinidae: *Abramisbrama*, *Alburnuschalcoides*, *Capoeta*, *Barbusbarbus*, *B.cyri*, *B.lacerta*, *Leuciscusleuciscus*, *Luciobarbusmursa*, *Romanogobiopersus*, *Rutilusrutilus*, *Varicorhinus* sp.

**Site of infection**: intestine.

##### Distribution

Occurring in Europe, Asia, North America; **in Georgia**: EG: Paravani Lake; Rivers: Alazani, Aragvi, Iori, Mtkvari; Khrami Reservoir; WG: River Mtkvari reported by Kurashvili et al. (1951), Kurashvili (1961a), Chiaberashvili (1962a), Chiaberashvili (1968), Kurashvili and Petriashvili (1977), Burtikashvili et al. (1978) and Kurashvili et al. (1980).

#### Allocreadium markewitschi

Koval, 1949

8BAEBACE-DA23-5A09-A83B-5B52BBB65E4F

##### Parasite of

fishes - Cyprinidae: *Alburnoides*, *Chondrostomacyri*.

**Site of infection**: intestine.

##### Distribution

Occurring in Europe; **in Georgia**: River Mtkvari; EG: River Alazani reported by Chiaberashvili (1968), Kurashvili et al. (1980) and Murvanidze et al. (2018).

#### Allocreadium transversale

(Rudolphi, 1802) Odhner, 1901

4FFA86A8-AD20-5BFC-9DC5-7486FB66AFFE

##### Parasite of

fishes - Cyprinidae: *Alburnusfilippii*, *Leuciscusleuciscus*, *Rutilusrutilus*.

**Site of infection**: intestine.

##### Distribution

Occurring in Europe, Asia; **in Georgia**: EG: Rivers: Iori, Mtkvari (surroundings of Borjomi, Mtskheta, Tbilisi, Khashuri); WG: River Rioni reported by Chiaberashvili (1962b), Chiaberashvili (1968), Kurashvili et al. (1980), Kurashvili et al. (1991) and Murvanidze et al. (2018).

#### Bunodera

Railliet, 1896

D23113E0-CCEC-5B2C-AAF9-F65CA707AD36

#### Bunodera luciopercae

(Müller, 1776) Lühe, 1909

DBDAC282-D91C-54FE-B1C1-96EF8915538C

##### Parasite of

fishes - Esocidae: *Esoxlucius*; Percidae: *Sanderlucioperca*; Siluridae: *Silurusglanis*.

**Site of infection**: intestine.

##### Distribution

Occurring in the Holarctic region; **in Georgia**: River Mtkvari reported by Kurashvili et al. (1980).

#### Crepidostomum

Braun, 1900

925A1B61-1E9F-5352-988B-49A7B57C5FE1

#### Crepidostomum farionis

(Müller, 1780) Lühe, 1909

B421B860-381A-5C7C-9599-A17DE3C52706

##### Parasite of

fishes - Cyprinidae: *Barbusbarbus*, *B.lacerta*, *Luciobarbusmursa*; Salmonidae: *Salmotruttafario*.

mollusc (intramolluscan stages) - Sphaeriidae: *Euglesacasertana*.

**Site of infection**: gall bladder, intestine.

##### Distribution

Occurring in the Holarctic; **in Georgia**: EG: Khrami Reservoir, Rivers: Alazani, Iori, Mtkvari (Borjomi Gorge), Ortachala, Khashuri; WG: Abkhazian coast of Black Sea, River Rioni reported by Kurashvili et al. (1951), Kurashvili (1961a), Chiaberashvili (1968), Chiaberashvili (1971a), Kurashvili et al. (1991) and Murvanidze et al. (2018).

#### Crepidostomum metoecus

(Braun, 1900) Braun, 1900

3BD336EA-348D-5BBF-B63E-63878798400D

##### Parasite of

molluscs (intramolluscan stage) - Sphaeriidae: *Pisidium* sp.

**Site of infection**: no data.

##### Distribution

Occurring in the Holarctic; **in Georgia**: WG: River Shavtskali reported by Chiaberashvili (1964).

#### Dicrocoeliidae

Looss, 1899

991E7A6F-A358-5DB7-BA59-0B68B6CCFBC0

#### Brachylecithum

Shtrom, 1940

503C88F5-808F-5229-B5D7-3B1C21EE2FFF

#### Brachylecithum attenuatum

(Dujardin, 1845) Shtrom, 1940

7F5CD336-803B-5937-A7AC-DE72DE3D396C

##### Parasite of

birds - Fringillidae: *Cardueliscarduelis*; Turdidae: *Turdusmerula*, *T.merulaatterimus*.

**Site of infection**: gallbladder.

##### Distribution

Occurring in Europe, Asia; **in Georgia**: EG: Lagodekhi National Park, Martkopi; WG: Sokhumi, surroundings of Samtredia reported by Kurashvili (1957), Kurashvili (1961a) and Kurashvili (1984b).

#### Corrigia

Shtrom, 1940

2BE5D8BB-A9DD-5CA3-BC74-CD547DE39AFE

#### Corrigia viktori

Gushanskaya, 1952

C8EA6605-4CE3-5D9C-937B-99DEF49934A7

##### Parasite of

birds - Phasianidae: *Coturnixcoturnix*.

**Site of infection**: small intestine.

##### Distribution

Recorded in Georgia only; **in Georgia**: WG: surroundings of Samtredia reported by Kurashvili (1957), Kurashvili (1961a) and Kurashvili (1984b).

#### Corrigia vitta

(Dujardin, 1845) Shtrom, 1940

FD71F2AF-13C8-5285-8402-04080D9FCEA1

##### Parasite of

mammals - Muridae: *Apodemussylvaticus*.

**Site of infection**: pancreas.

##### Distribution

Occurring in Europe; **in Georgia**: EG: Borjomi reported by Matsaberidze (1966a), Matsaberidze (1966b) and Matsabaridze (1976).

#### Dicrocoelium

Dujardin, 1845

3DC89567-BF0F-5F83-B397-6E58E37B6664

#### Dicrocoelium dendriticum

(Rudolphi, 1819) Looss, 1899

96DE2716-2327-50EE-A211-5B3F9FB93790

Dicrocoelium lanceatum Stiles & Hassall, 1898

##### Parasite of

mammals - Bovidae: *Bostaurus*, *Caprahircus*, *Ovisaries*; Cervidae: *Cervus*, *Capreoluscapreolus*; Leporidae: *Lepus*; Suidae: *Susscrofa*; Ursidae: *Ursusarctos*; Muridae: *Chionomys*.

molluscs (intramolluscan stage) - Enidae: *Georginapaeushohenackeri*; Geomitridae: *Xeropictaderbentina*; Hygromiidae: *Harmozicaravergiensis*.

**Site of infection**: gallbladder, hepatic ducts, liver.

##### Distribution

Cosmopolitan distribution; **in Georgia**: EG: Akhmeta, Borjomi, Dedoflistskaro, Dusheti, Kazbegi, Lagodekhi, Tianeti, Highland regions of southern and northern Georgia; WG: Akhalqalaqi, Akhaltsikhe, Batumi, Khobi, Lentekhi, Samtredia, Tsalenjikha, Zugdidi reported by Burdjanadze (1937b), Gamtsemlidze (1941), Kurashvili and Rodonaia (1954), Qoiava (1956a), Qoiava (1961), Kurashvili (1961a), Rodonaia (1962), Rodonaia (1966a), Rodonaia (1966b), Natsvlishvili (1968), Rodonaia (1971) and Matsabaridze (1976).

#### Dicrocoelium macrostomum

Odhner, 1910

3CFF0E91-AFB5-5CD7-83A4-1EC6383BB462

##### Parasite of

birds - Phasianidae: *Coturnixcoturnix*; Numididae: *Numidameleagris*.

**Site of infection**: hepatic ducts, liver.

##### Distribution

Occurring in Africa; **in Georgia**: WG: Samtredia reported by Kurashvili (1957), Kurashvili (1961a) and Kurashvili (1984b).

#### Dicrocoelium sp.

AC763C62-6856-5943-B608-05F6FB570AB4

##### Parasite of

molluscs (intramolluscan stage) - Oxychilidae: *Oxychilusmingrelicus*.

**Site of infection**: hepatopancreas.

##### Distribution

Cosmopolitan distribution; **in Georgia**: **WG**: Samegrelo reported by Arabuli (2018).

#### Eurytrema

Looss, 1907

DE812431-AE4C-55DB-8B54-3543A78EDF0C

#### Eurytrema pancreaticum

(Janson, 1889) Looss, 1907

EFEE6E27-D30E-57D5-BDB8-A0510B190928

##### Parasite of

mammals - Bovidae: *Bostaurus*.

**Site of infection**: liver.

##### Distribution

Occurring in Europe, Madagascar, Asia, and South America; **in Georgia**: WG: lowland of Kolkheti reported by Burjanadze (1943) and Kurashvili (1984b).

#### Lyperosomum

Looss, 1899

945D77AB-B635-503E-9A2A-B135CEBFBF64

#### Lyperosomum petiolatum

(Railliet, 1900)

DB6F5357-CC5B-5A3D-9741-687361439696

Skrjabinus popovi Kassimov, 1952
Zonorchis petiolatum (Railliet, 1900)

##### Parasite of

birds - Phasianidae: *Tetraogalluscaucasicus*.

**Site of infection**: gallbladder, liver.

##### Distribution

Occurring in Europe; **in Georgia**: EG: Lagodekhi National Park reported by Kurashvili (1957).

#### Platynosomum

Looss, 1907

B32D7521-248A-5F06-9B47-864F47AE6EC9

#### Platynosomum fallax

Heidegger & Mendheim, 1938

8953CDC1-F791-5DFB-9004-DA93DE464954

##### Parasite of

birds - Picidae: *Picusviridiskarelini*.

**Site of infection**: gallbladder, liver.

##### Distribution

Occurring in Asia; **in Georgia**: EG: Lagodekhi National Park, Telavi – Alazani River; WG: surroundings of Samtredia reported by Kurashvili (1957), Kurashvili (1961a) and Kurashvili (1984b).

#### Encyclometridae

Mehra, 1931

BD084946-49A2-5324-9DD5-33E58B305283

#### Encyclometra

Baylis & Cannon, 1924

347B7511-9766-5562-AD14-F92E20A912FB

#### Encyclometra colubrimurorum

(Rudolphi, 1891) Dollfus, 1931

0BB1735F-A005-595C-AAD6-9E25678CAA74

##### Parasite of

reptiles - Colubridae: *Natrixnatrix*.

**Site of infection**: oesophagus, intestine, stomach.

##### Distribution

Palaearctic distribution; **in Georgia**: EG: Jinvali, Kazbegi, surroundings of Tbilisi; WG: surroundins of Batumi – Kakhaberi Lake, Anaklia, Kulevi, Khobi, Zugdidi reported by Kurashvili (1984b) and Jankarashvili (1985).

#### Gorgoderidae

Looss, 1899

92BE2256-6EC8-5E5B-A2D0-DCA9DDEB214D

#### Gorgodera

Looss, 1899

9039BDD6-12CF-5202-AF77-7E6C553974F6

#### Gorgodera asiatica

Pigulewski, 1943

1F8C6323-2834-5156-A867-22E5E8B1916B

##### Parasite of

amphibians - Ranidae: *Pelophylaxridibundus*, *Ranamacrocnemis*.

**Site of infection**: urinary bladder.

##### Distribution

Palaearctic distribution; **in Georgia**: EG: Akhaldaba, Borjomi, Aragvi River, Lakes: Bazaleti, Jandari Lake, Kumisi; WG: Ozurgeti, Tkibuli Reservoir reported by Chiaberashvili and Mchedlidze (1961), Kurashvili et al. (1973), Kurashvili et al. (1975), Burtikashvili and Getzadze (1981), Giorgadze (1985), Petriashvili et al. (1985), Kurashvili et al. (1991) and Murvanidze et al. (2008a).

#### Gorgodera cygnoides

(Zeder, 1800) Looss, 1899

96048E7A-A0F9-58FE-B1DE-472755820107

##### Parasite of

amphibians - Bufonidae: *Bufotesviridis*, Ranidae: *Pelophylaxridibundus*, *Ranamacrocnemis*.

**Site of infection**: urinary bladder.

##### Distribution

Holarctic and Australasian distribution; **in Georgia**: EG: Bazaleti Lake, Kumisi Reservoir, Tbilisi Reservoir; WG: Khobi, Ozurgeti, Senaki, Tkibuli Reservoir reported by Chiaberashvili and Mchedlidze (1961), Petriashvili (1964), Kurashvili (1984b), Giorgadze (1985), Kurashvili et al. (1991) and Murvanidze et al. (2008a).

#### Gorgodera dollfusi

Pigulewsky, 1946

B8CD7314-5EC5-5C08-A5E6-A63EFE119B8B

##### Parasite of

amphibians - Bufonidae: *Bufobufo*, *Bufotesviridis*; Ranidae: *Pelophylaxridibundus*.

**Site of infection**: urinary bladder.

##### Distribution

Occurring in Asia; **in Georgia**: EG: Aragvi River, Martkhopi reported by Kurashvili et al. (1977), Burtikashvili et al. (1978), Burtikashvili and Getzadze (1981), Petriashvili et al. (1985) and Murvanidze et al. (2008a).

#### Gorgodera pagenstecheri

Sinizin, 1905

EBC57BF0-7BFE-5A00-B62E-233AA858E70C

##### Parasite of

amphibians - Ranidae: *Pelophylaxridibundus*, *Ranamacrocnemis*.

molluscs (intramolluscan stage) - Sphaeriidae: *Sphaeriumcorneum*.

**Site of infection**: urinary bladder.

##### Distribution

Occurring in Europe; **in Georgia**: EG: Akhaldaba, Kodjori, Tbilisi Botanic Garden; WG: Ozurgeti, Sukhumi, Tkibuli Reservoir reported by Chiaberashvili and Mchedlidze (1961), Kurashvili (1984b), Giorgadze (1985), Petriashvili et al. (1985), Kurashvili et al. (1991) and Murvanidze et al. (2008a).

#### Gorgoderina

Looss, 1902

8090303F-9803-5418-A6FD-C82BED03A782

#### Gorgoderina vitelliloba

(Olsson, 1876) Looss, 1902

9889446C-1789-59DC-8E1A-3DCBB19D0CDE

##### Parasite of

amphibians - Ranidae: *Pelophylaxridibundus*, *Ranamacrocnemis*.

molluscs (intramolluscan stage) - Sphaeriidae: *Euglesacasertana*, *Sphaeriumcorneum*.

**Site of infection**: hepatopancreas, urinary bladder.

##### Distribution

With Palaearctic distribution; **in Georgia**: EG: Jandari Lake, Kazbegi, Tbilisi – Samgori; WG: Bebesiri Lake, Khobi, Ozurgeti, Samtredia, Tkibuli Reservoir reported by Chiaberashvili and Mchedlidze (1961), Chiaberashvili (1971a), Kurashvili et al. (1975), Kurashvili (1984a), Kurashvili (1984b), Giorgadze (1985), Petriashvili et al. (1985) and Murvanidze et al. (2008a).

#### Phyllodistomum

Braun, 1899

5547EEEF-D402-52F5-A69A-DFE21344A5CB

#### Phyllodistomum folium

(Olfers, 1816) Braun, 1899

3EFF290B-54AA-55B5-9D05-FAE9B211A0F7

Phyllodistomum elongatum Nybelin, 1926

##### Parasite of

fishes - Cyprinidae: *Abramisbrama*, *Alburnuschalcoides*, *Barbusbarbus*, *Luciobarbuscapito*, *Rutilusrutilus*, *Scardiniuserythrophthalmus*, *Squaliuscephalus*, *Vimbavimba*.

**Site of infection**: urinary bladder.

##### Distribution

Palaearctic distribution; **in Georgia**: EG: River Mtkvari; WG: Rivers: Mtkvari, Rioni, Tekhura; Bebesiri Lake reported by Chiaberashvili (1962b), Kurashvili et al. (1980) and Murvanidze et al. (2018).

#### Phyllodistomum pseudofolium

Nybelin, 1926

F99A3F1F-B32A-5EA5-AB41-3F95657314F0

##### Parasite of

fishes - Cyprinidae: *Alburnusderjugini*, *Chondrostomacolchicum*, *Vimbavimba*.

**Site of infection**: urinary bladder.

##### Distribution

Occurring in Europe; **in Georgia**: WG: Bebesiri Lake, River Rioni reported by Chiaberashvili (1962b) and Murvanidze et al. (2018).

#### Haploporoidea

Nicoll, 1914

66E0A4BF-92A9-5764-9ADC-203DB1A4DAFA

#### Haploporidae

Nicoll, 1914

2D841075-218B-5759-B7F6-9FF6221E57CC

#### Saccocoelium

Looss, 1902

70FC23E8-9315-5C76-86D3-CA2891867D51

#### Saccocoelium obesum

Looss, 1902

141E4388-613C-548A-BA08-956E5F599F00

##### Parasite of

fishes - Mugilidae: *Chelonauratus*, *Mugilcephalus*.

**Site of infection**: intestine.

##### Distribution

Occurring in Europe; **in Georgia**: WG: Paliastomi Lake reported by Chernova (1973) and Murvanidze et al. (2018).

#### Haplosplanchnoidea

Poche, 1925

0ABB7EEB-EB34-555D-9346-691C7A914C81

#### Haplosplanchnidae

Poche, 1926

9447D06C-1A2A-51FD-88AB-CA12D7FDA6E6

#### Haplosplanchnus

Looss, 1902

0BF7F0B6-A42E-5AC6-9246-104D83B8CE76

#### Haplosplanchnus pachysoma

(Eysenhardt, 1829) Looss, 1902

CCC26575-AD7D-538D-9D7E-F4FEDFA2210E

##### Parasite of

fishes - Mugilidae: *Chelonauratus*, *Mugilcephalus*.

**Site of infection**: intestine.

##### Distribution

Occurring in Europe, Africa, Oceanian Regions; **in Georgia**: WG: Paliastomi Lake reported by Chernova (1973) and Murvanidze et al. (2018).

#### Schikhobalotrema

Skrjabin & Guschanskaya, 1955

57213422-F215-5D36-8C46-B702C223DBFB

#### Schikhobalotrema sparisomae

(Manter, 1938) Skrjabin & Guschanskaya, 1955

33AD67F7-8CAC-5BB8-BD35-8CBCD39A481C

##### Parasite of

fishes - Mugilidae: *Chelonauratus*.

**Site of infection**: digestive tract.

##### Distribution

Holarctic distribution; **in Georgia**: WG: Paliastomi Lake reported by Chernova (1973) and Murvanidze et al. (2018).

#### Hemiuroidea

Looss, 1899

976ADBC0-1640-545D-806A-442FE16D4ABC

#### Hemiuridae

Looss, 1899

EE184988-F5AD-5480-86D7-8FAD9B213E69

#### Aphanurus

Looss, 1907

E95F1629-E4DF-5019-A5B5-C82269DEB038

#### Aphanurus stossichii

(Monticelli, 1891) Looss, 1907

BAC4C7B0-9179-55FC-A7F0-DF10A8B54BF3

##### Parasite of

fishes - Clupeidae: *Alosatanaica*.

**Site of infection**: no data.

##### Distribution

Palaearctic distribution; **in Georgia**: WG: Paliastomi Lake reported by Chernova (1973).

#### Hemiurus

Rudolphi, 1809

D40ACF3A-F7BF-59C4-8C16-FD0F879EC920

#### Hemiurus appendiculatus

(Rudolphi, 1802) Looss, 1899

C84C96F2-5538-5165-8B3C-E5D22DA3DD31

##### Parasite of

fishes - Clupeidae: *Alosatanaica*, *A.immaculata*; Mugilidae: *Chelonauratus*, *Mugilcephalus*; Percidae: *Percafluviatilis*.

**Site of infection**: stomach.

##### Distribution

Occurring in Europe, North America, North Africa; **in Georgia**: WG: Paliastomi Lake reported by Kurashvili et al. (1951), Kurashvili (1961a), Kurashvili and Petriashvili (1977) and Murvanidze et al. (2018).

#### Hemiurus luehei

Odhner, 1905

1454E093-ED69-5164-A203-0C97E1E4FFA2

##### Parasite of

fishes - Clupeidae: *Alosaimmaculata*.

**Site of infection**: stomach.

##### Distribution

Holarctic distribution; **in Georgia**: WG: Sokhumi reported by Kurashvili and Tabidze (1947).

#### Lecithasteridae

Odhner, 1905

56AD0213-4A31-5C85-B1EE-8FBE46D13B55

#### Aponurus

Looss, 1907

B3F1CB93-C076-5ACE-9240-EE5B1827B8B4

#### Aponurus tschugunovi

Issaitschikov, 1927

AD86844C-B795-531D-814B-9225AFAF0A4C

##### Parasite of

fishes - Mugilidae: *Mugilcephalus*.

**Site of infection**: no data

##### Distribution

Occurring in the Black sea, European part of Russia; **in Georgia**: WG: Paliastomi Lake reported by Chernova (1973).

#### Lecithaster

Lühe, 1901

ECCDE730-87A5-5A44-96FB-8F76AD8CE901

#### Lecithaster confusus

Odhner, 1905

373673C5-CC75-5774-80B8-B91D6F5E001F

Lecithaster musteli Srivastava, 1966

##### Parasite of

fishes - Cyprinidae: *Alburnusderjugini*; Clupeidae: *Alosaimmaculata*.

**Site of infection**: intestine.

##### Distribution

Occurring in the Holarctic Region, Africa; **in Georgia**: WG: Paliastomi Lake reported by Chernova (1977) and Murvanidze et al. (2018).

#### Lecithaster tauricus

Pigulewsky, 1938

066C9FBF-771D-5FE2-A41C-F7327D9E626D

##### Parasite of

fishes - Esocidae: *Esoxlucius*; Percidae: *Percafluviatilis*.

**Site of infection**: intestine.

##### Distribution

Occurring in the Black Sea; **in Georgia**: WG: Paliastomi Lake reported by Chernova (1977) and Murvanidze et al. (2018).

#### Microphalloidea

Ward, 1901

FDFD1AD8-886B-55C8-BA05-12C3A044446D

#### Eucotylidae

Skrjabin, 1924

86DF6428-830E-52E9-8D3F-D5E29CD7FB6E

#### Eucotyle

Cohn, 1904

CD3301B1-9571-555E-9499-C5D921E64B47

#### Eucotyle popowi

Skrjabin & Evramova, 1942

B351E0E4-81CE-5028-A7B0-E0FAB29ED528

##### Parasite of

birds - Anatidae: *Anasplatyrhynchos*, *Melanittafusca*; Podicipedidae: *Podicepscristatus*.

**Site of infection**: ureter, urethra.

##### Distribution

Holarctic distribution; **in Georgia**: WG: Batumi, Paliastomi Lake, Poti reported by Kurashvili (1961b), Kurashvili et al. (1976) and Kurashvili (1984b).

#### Neoeucotyle

Kanev, Radev & Fried, 2002

872686DB-2707-5EA7-9563-6E8EE133004A

#### Neoeucotyle zakharowi

(Skrjabin, 1920) Kanev, Radev & Fried, 2002

A591568E-74A3-5737-88F7-066FC83711E4

Eucotyle zakharowi Skrjabin, 1920

##### Parasite of

birds - Anatidae: *Anaspenelope*, *A.plathyrynchos*, A.plathyrynchosf.domestica, *A.querquedula*.

**Site of infection**: kidney, ureter, urethra.

##### Distribution

Occurrence in Palaearctic Region; **in Georgia**: EG: Dusheti – Bazaleti Lake, Gardabani, surroundings of Tbilisi Sea, Tetritskaro, Tsalka; WG: Samtredia reported by Kurashvili et al. (1976).

#### Tamerlania

Skrjabin, 1924

BE887AEC-5E1B-582C-B200-47E8EF97CD95

#### Tamerlania zarudnyi

Skrjabin, 1924

7EE7CBE7-4DC2-592C-8F4C-C497C22CA265

##### Parasite of

birds - Passeridae: *Passerdomesticus*.

**Site of infection**: kidneys, ureter, urethra.

##### Distribution

Occurring in the Holarctic, Madagascar; **in Georgia**: EG: surroundings of Tbilisi reported by Kurashvili (1941), Kurashvili (1957), Kurashvili (1961a) and Kurashvili et al. (1976).

#### Eumegacetidae

Travassos, 1922

18D9F205-72D5-57DC-85DD-5CF9B635F350

#### Eumegacetes

Looss, 1900

0F845202-2238-501E-B851-5FC604B3A55B

#### Eumegacetes ibericus

Kurashvili, 1941

FBD10383-B4E8-5BB8-AC05-E680BB111AA0

##### Parasite of

birds - Passeridae: *Passerdomesticus*.

**Site of infection**: large intestine.

##### Distribution

Occurring in Europe; **in Georgia**: EG: surroundings of Tbilisi reported by Kurashvili (1940), Kurashvili (1941), Kurashvili (1957) and Kurashvili (1961a).

#### Faustulidae

Poche, 1926

49F5D50E-4747-5095-9DEF-F4718DDF6089

#### Pronoprymna

Poche, 1926

E1B5F234-C075-51E9-8679-40C159D87CCB

#### Pronoprymna ventricosa

(Rudolphi, 1819) Poche, 1926

611E71FE-923F-5B19-A7B0-6047856AD9DE

Pentagramma symmetricum Chulkova, 1939

##### Parasite of

Fish - Clupeidae: *Alosatanaica*, *A.immaculata*.

**Site of infection**: no data.

##### Distribution

Occurring in the Palaearctic; **in Georgia**: WG: Paliastomi Lake reported by Chernova (1973).

#### Lecithodendriidae

Lühe, 1901

884C8863-A298-5424-B675-5A53855AB784

#### Lecithodendrium

Looss, 1896

8821CBBA-BDF0-57BA-B342-C93B2C184631

#### Lecithodendrium dryomi

Mazaberidze & Chotenovskiy, 1966

8BD7D92D-E81D-52D7-9E1B-AAD4A74A0A1D

##### Parasite of

mammals - Gliridae: *Dryomysnitedula*; Myoxidae.

**Site of infection**: small intestine.

##### Distribution

Occurrence recorded in Georgia only; **in Georgia**: EG: Borjomi – Gujareti reported by Matsaberidze (1966b), Matsaberidze and Khotenovskii (1966b), Matsaberidze and Khotenovskii (1967) and Matsabaridze (1976).

#### Lecithodendrium linstowi

Dollfus, 1931

A9C99442-B9B5-5330-B0AB-188B3EEA8DC6

##### Parasite of

mammals - Vespertilionidae: *Eptesicusserotinus*, *Pipistrellusnathusii*.

**Site of infection**: small intestine.

##### Distribution

Occurring in Europe; **in Georgia**: EG: Aspindza, Borjomi, Dmanisi, Khashuri, Lagodekhi, Sagarejo; WG: Tkibuli reported by Matsaberidze (1966b), Matsaberidze and Khotenovskii (1967) and Matsabaridze (1976).

#### Lecithodendrium rysavyi

Dubois, 1960

EF155E61-70D6-56D4-8B78-D091AF31F451

##### Parasite of

mammals - Vespertilionidae: *Pipistrelluskuhlii*.

**Site of infection**: small intestine.

##### Distribution

Occurring in Europe; **in Georgia**: EG: Tbilisi; WG: Tsalenjikha reported by Matsaberidze and Khotenovskii (1967) and Matsabaridze (1976).

#### Lecithodendrium semen

Kirschenblatt, 1941

5743672F-7B66-5FD3-A2BA-A12C26335A1F

##### Parasite of

mammals - Gliridae: *Dryomisnitedula*.

**Site of infection**: small intestine.

##### Distribution

Recorded in Georgia only; **in Georgia**: WG: Bakhmaro, Chokhatauri reported by Kirshenblat (1941), Kurashvili (1961a), Rodonaia (1971) and Matsabaridze (1976).

#### Lecithodendrium skrjabini

Matsaberidze, 1963

033F3F6F-89E9-5744-8B8B-5D370070565B

##### Parasite of

mammals - Vespertilionidae: *Pipistrellusnathusii*.

**Site of infection**: small intestine.

##### Distribution

Occurring in Europe; **in Georgia**: WG: Tkibuli reported by Matsaberidze (1963), Matsaberidze and Khotenovskii (1967) and Matsabaridze (1976).

#### Ophiosacculus

Macy, 1935

43347AE4-8E99-527D-93C0-CAB76979D1C7

#### Ophiosacculus mehelyi

(Mödlinger, 1930) Macy, 1935

2B4F9C68-33A3-522A-9C67-205FE53796A5

Ophiosacculus eptesicus Matsaberidze & Chotenowsky, 1965

##### Parasite of

mammals - Vespertilionidae: *Eptesicusserotinus*.

**Site of infection**: small intestine.

##### Distribution

Occurring in Europe; **in Georgia**: EG: Mtskheta, Sagarejo – Gombori, Tbilisi reported by Matsaberidze (1966b), Matsaberidze and Khotenovskii (1966a), Matsaberidze and Khotenovskii (1967), Matsabaridze (1976) and Tkach et al. (2002).

#### Prosotocus

Looss, 1899

97593C27-33E4-5E82-8EDD-75923EC72488

#### Prosotocus confusus

(Loos, 1896) Looss, 1899

32A7379D-319E-544C-8B3C-127CC6E04F6A

##### Parasite of

amphibians - Ranidae: *Pelophylaxridibundus*, *Ranamacrocnemis*.

**Site of infection**: small intestine.

##### Distribution

With Palaearctic distribution; **in Georgia**: WG: Ozurgeti, Samtredia, Senaki reported by Chiaberashvili and Mchedlidze (1961) and Kurashvili (1984b).

#### Prosotocus fuelleborni

Travassos, 1930

A683C211-9B06-5225-9E12-2F19A45ACAF1

##### Parasite of

amphibians - Ranidae: *Pelophylaxridibundus*.

**Site of infection**: small intestine.

##### Distribution

Occurring in Europe; **in Georgia**: EG: Lakes: Bazaleti, Kumisi reported by Petriashvili (1964) and Kurashvili et al. (1973).

#### Prosthodendrium

Dollfus, 1931

12A90D3A-276A-5333-A926-ABDE014A2DDE

#### Prosthodendrium ascidia

(van Beneden, 1873) Bhalerao, 1936

8212BC8B-FC78-53C8-84A9-D6ADE2D2AF96

##### Parasite of

mammals - Vespertilionidae: *Eptesicusserotinus*, *Pipistrelluskuhlii*, *P.nathusii*, Rhinolophidae: *Rhinolophusferrumequinum*.

**Site of infection**: small intestine.

##### Distribution

Occurring in Europe; **in Georgia**: EG: Dedoflistskaro – Shiraqi, Gareji, Sagarejo-Gombori; WG: Tkibuli reported by Matsaberidze and Khotenovskii (1967) and Matsabaridze (1976).

#### Prosthodendrium chilostomum

(Mehlis, 1831) Travassos, 1921

06BF1565-653D-5DAB-9220-25AB84CD825E

##### Parasite of

mammals - Vespertilionidae: *Eptesicusserotinus*, *Pipistrellusnathusii*; Rhinolophidae: *Rhinolophusferrumequinum*.

**Site of infection**: small intestine.

##### Distribution

Occurring in Europe; **in Georgia**: EG: Mtskheta, Natakhtari, Sagarejo-Gombori; WG: Tkibuli reported by Matsaberidze and Khotenovskii (1967) and Matsabaridze (1976).

#### Prosthodendrium parvouterus

(Bhalerao, 1926) Skrjabilovich, 1948

5AFACF19-2CAF-50C8-A250-0F489CFBEEAD

##### Parasite of

mammals - Vespertilionidae: *Eptesicusserotinus*, *Nyctalusnoctula*.

**Site of infection**: small intestine.

##### Distribution

Occurring in the Holarctic Region, Africa; **in Georgia**: EG: Borjomi, Dedoflistskaro – Shiraqi, Sagarejo-Gombori; WG: Tkibuli reported by Matsaberidze and Khotenovskii (1967) and Matsabaridze (1976).

#### Prosthodendrium skrjabini

(Shaldybin in Skarbilovich, 1948)

1B504BF4-885E-505F-B4D5-51D1BF9DF222

Paralecithodendrium skrjabini Shaldybin, 1948

##### Parasite of

mammals - Vespertilionidae: *Eptesicusserotinus*; Rhinolophidae: *Rhinolophusferrumequinum*.

**Site of infection**: intestine.

##### Distribution

Occurring in Europe; **in Georgia**: EG: Borjomi, Davit Gareji reported by Matsaberidze (1966b) and Matsabaridze (1976).

#### Pycnoporus

Looss, 1899

2E732205-413A-511C-891D-4176C50C90FC

#### Pycnoporus kuraschvili

Matzaberidse, 1976

667DFEF7-7621-52E0-B4BC-F9C8DA055288

##### Parasite of

mammals - Vespertilionidae: *Myotismistacinus*.

**Site of infection**: small intestine.

##### Distribution

Recorded in Georgia only; **in Georgia**: EG: Tbilisi reported by Matsabaridze (1976).

#### Phaneropsolidae

Mehra, 1935

A07AF824-A751-502C-9085-288C8241BBB3

#### Combesia

Mas-Coma, Roset & Montoliu, 1985

1C62B81F-4ACF-5622-89C2-57E36C5BE9DE

#### Combesia macrobursata

(Tschertkova & Rodonaja, 1965) Mas-Coma, Roset & Montoliu, 1985

F2788C97-CE70-5120-BA49-20282DB121EF

Plagiorchis macrobursatum Tschertkova & Rodonaja, 1965

##### Parasite of

mammals - Talpidae: *Talpaeuropea*, *T.orientalis*.

**Site of infection**: small intestine.

##### Distribution

Recorded from Georgia and Spain; **in Georgia**: WG: Sokhumi reported by Chertkova and Rodonaia (1965), Rodonaia (1971) and Matsabaridze (1976).

#### Pleurogenidae

Looss, 1899

58E43725-4B7C-504C-8165-548693981604

#### Pleurogenes

Looss, 1896

93201626-2B23-5BCD-8F1D-296F4BF0BB5F

#### Pleurogenes claviger

(Rudolphi, 1819) Looss, 1896

4DF5FB81-3BBE-5F9D-9523-7FC824FED1FF

##### Parasite of

amphibians - Ranidae: *Pelophylaxridibundus*, *Ranamacrocnemis*.

**Site of infection**: intestine.

##### Distribution

Occurring in Europe; **in Georgia**: WG: Bebesiri Lake, Gali, Gudauta reported by Chiaberashvili and Mchedlidze (1961), Kurashvili (1984b), Petriashvili et al. (1985) and Murvanidze et al. (2008a).

#### Pleurogenes intermedius

Issaitschikov, 1926

D7FC3512-0363-5DAB-93FE-66DFE103FB4A

##### Parasite of

amphibians - Bufonidae: *Bufotesviridis*; Ranidae: *Pelophylaxridibundus*, *Ranamacrocnemis*.

**Site of infection**: intestine.

##### Distribution

Palaearctic distribution; **in Georgia**: EG: Akhaldaba, Borjomi reported by Kurashvili et al. (1991) and Murvanidze et al. (2008a).

#### Pleurogenoides

Travassos, 1921

BDBE8E8C-5091-5271-8452-70F27DA4FBBD

#### Pleurogenoides medians

(Olsson, 1876) Travasos, 1921

37B4C573-76A7-5A00-A507-03FAABAA9660

##### Parasite of

amphibians - Bufonidae: *Bufotesviridis*; Ranidae: *Pelophylaxridibundus*, *Ranamacrocnemis*.

**Site of infection**: small intestine.

##### Distribution

Holarctic distribution; **in Georgia**: EG: Aragvi River Basin, Bazaleti Lake, Martkhopi; WG: Gali, Khobi reported by Petriashvili (1964), Kurashvili et al. (1977), Burtikashvili and Getzadze (1981), Petriashvili et al. (1985) and Murvanidze et al. (2008a).

#### Prosthogonimidae

Lühe, 1909

4345AAC8-4BE6-5197-A9EC-A3C192F3FD6F

#### Prosthogonimus

Lühe, 1899

86C39E7C-1F2D-5F9E-BD34-C503193E5D35

#### Prosthogonimus ovatus

(Rudolphi, 1803) Lühe, 1899

CA95B974-3F3C-5DA7-B0C1-D7E2680B5E98

##### Parasite of

birds - Anatidae: *Anasplathyrynchos*, A.platyrhynchosf.domestica, *A.strepera*, *Anseranser*; Phasianidae: Gallusgallusf.domestica, *Phasianuscolchicus*.

molluscs (intramolluscan stages) - Bithyniidae: *Bithyniatentaculata*.

**Site of infection**: bursa Fabricii, oviduct.

##### Distribution

Occurring in Europe, Asia, Americas; **in Georgia**: EG: Dusheti – Bazaleti Lake, Gardabani, Lisi Lake, Tetritskaro, Tsalka; WG: Bebesiri Lake, Gagra, Samtredia reported by Burjanadze (1943), Kurashvili (1957), Kurashvili (1961a), Japaridze and Savateeva (1967), Kurashvili et al. (1976), Kurashvili (1984a) and Kurashvili (1984b).

#### Stomylotrematidae

Poche, 1926

B6D9C2AD-834F-5571-A12D-D44B87D0409E

#### Stomylotrema

Looss, 1900

8184B27C-C1E4-527F-9576-7C407993C2BA

#### Stomylotrema spasskii

Sobolev, 1946

C1559091-722C-53FC-83C9-3685A47B13AA

##### Parasite of

birds - Scolopacidae: *Gallinagomedia*.

**Site of infection**: bursa Fabricii, large intestine.

##### Distribution

Occurring in Europe; **in Georgia**: WG: Sokhumi reported by Kurashvili (1950), Kurashvili (1953a), Kurashvili (1957), Kurashvili (1961a) and Kurashvili (1984b).

#### Monorchioidea

Odhner, 1911

9BA4946D-3110-50B4-B6E5-225D2D427FD5

#### Deropristidae

Cable & Hunninen, 1942

1ED6604B-4934-5018-8AB2-D7D815513B75

#### Skrjabinopsolus

Ivanov in Ivanov & Murygin, 1937

49C00A34-2B50-5C0D-B051-AB56522523BB

#### Skrjabinopsolus semiarmatus

(Molin, 1858) Ivanov in Ivanov & Murygin, 1937

596BFE03-1A5F-553C-AE8C-F680B8F6BF1C

Skrjabinopsolus acipenseris Ivanov & Murygin, 1937
Skrjabinopsolus minor Bychowskaya-Pavlovskaya & Mikailov, 1969

##### Parasite of

fishes - Acipenseridae: Acipenserpersicus, *A.stellatus*, *Husohuso*.

**Site of infection**: oesophagus, intestine, stomach.

##### Distribution

Occurring in the Holarctic; **in Georgia**: River Mtkvari; WG: River Rioni (Orpiri Village), surroundings of Batumi reported by Chulkova (1939), Kurashvili et al. (1980) and Murvanidze et al. (2018).

#### Lissorchiidae

Magath, 1917

F9D766A4-BE7C-5023-9C61-25FE05840805

#### Asymphylodora

Looss, 1899

BE3CD52F-B50E-5041-A544-83FF60AC7A71

#### Asymphylodora demeli

Markowski, 1935

16EA2261-0AD9-5FC0-92E2-B934D86A99CF

##### Parasite of

fishes - Gobiidae: *Ponticolaconstructor*.

**Site of infection**: intestine.

##### Distribution

Occurring in Europe; **in Georgia**: River Mtkvari reported by Kurashvili et al. 1980.

#### Asymphylodora kubanica

Issaitschikov, 1923

44E8E8EA-C038-549D-9D37-D96686D14674

##### Parasite of

fishes - Cyprinidae: *Abramisbrama*, *Alburnuschalcoides*, *Ballerussapa*, *Bliccabjoerkna*, *Cyprinuscarpio*, *Leuciscusaspius*, *Rutilusrutilus*; Esocidae: *Esoxlucius*; Siluridae: *Silurusglanis*.

**Site of infection**: intestine.

##### Distribution

Occurring in Europe, Asia; **in Georgia**: River Mtkvari reported by Kurashvili et al. (1980).

#### Asymphylodora markewitschi

Kulakowskaya, 1947

280E487E-E9E3-5200-B975-7619348AC275

##### Parasite of

fishes - Cyprinidae: *Barbuslacerta*, *Cyprinuscarpio*.

**Site of infection**: oesophagus, intestine.

##### Distribution

Occurring in Europe, Asia; **in Georgia**: EG: River Mtkvari reported by Kurashvili et al. (1980) and Kurashvili et al. (2005).

#### Asymphylodora tincae

(Modeer, 1790) Lühe, 1909

CA29F7E3-8498-565A-AC91-88B0A44FDF7E

##### Parasite of

fishes - Clupeidae: *Alosatanaica*, *A.immaculata*; Cyprinidae: *Luciobarbuscapito*, *Tincatinca*; Esocidae: *Esoxlucius*; Mugilidae: *Mugilcephalus*.

**Site of infection**: oesophagus, intestine.

##### Distribution

Occurring in Europe; **in Georgia**: River Mtkvari; WG: Lakes: Japana, Paliastomi reported by Chernova (1973), Kurashvili et al. (1980) and Murvanidze et al. (2018).

#### Opecoeloidea

Ozaki, 1925

C4054DB6-CE1B-5D29-9A32-17746C6C5226

#### Opecoelidae

Ozaki, 1925

F1926B71-AEE8-52B7-A97A-B57866C9658A

#### Sphaerostoma

Rudolphi, 1809

4BD94843-7DB6-57B2-83FF-A9DCC71959DD

#### Sphaerostoma bramae

(Müller, 1776) Lühe, 1909

DABAEDAF-A4A8-5710-94C1-BE350991FD77

##### Parasite of

fishes - Cyprinidae: *Alburnuschalcoides*, *Ballerussapa*, *Bliccabjoerkna*; Esocidae: *Esoxlucius*.

**Site of infection**: intestine.

##### Distribution

Palaearctic distribution; **in Georgia**: River Mtkvari reported by Kurashvili et al. (1980).

#### Sphaerostoma globiporum

(Rudolphi, 1802) Looss, 1899

6F07C541-4DFD-510B-B2A6-CDA0E059E973

##### Parasite of

fishes - Cyprinidae: *Rutilusrutilus*.

**Site of infection**: intestine.

##### Distribution

Occurrence in Europe, North America; **in Georgia**: WG: Paliastomi Lake reported by Chernova (1973) and Murvanidze et al. (2018).

#### Sphaerostoma salmonis

Slusarski, 1958

15FEC2DF-F422-58EB-B968-5A1E0E690760

Sphaerostoma kurensis Mikailov, 1969

##### Parasite of

fishes - Cyprinidae: *Alburnuschalcoides*.

**Site of infection**: intestine.

##### Distribution

Occurring in Europe; **in Georgia**: River Mtkvari reported by Kurashvili et al. (1980).

#### Opisthorchioidea

Looss, 1899

0D4378DA-973E-5C6F-95AA-607E4213B35E

#### Cryptogonimidae

Ward, 1917

AD30C944-8008-5389-AC36-AC8070312D45

#### Timoniella

Rebecq, 1960

AF5F7653-90AB-5771-B911-433C1182FD14

#### Timoniella imbutiformis

(Molin, 1859) Brooks, 1980

E50031E0-252D-50C7-9262-F275BFF0502E

##### Parasite of

fishes - Clupeidae: *Alosatanaica*.

**Site of infection**: no data.

##### Distribution

Occurring in the Mediterranean Region, Caspian Sea, Black Sea, northeast Atlantic; **in Georgia**: WG: Paliastomi Lake reported by Burjanadze (1943) and Murvanidze et al. (2018).

#### Heterophyidae

Leiper, 1909

97E38E2C-96B9-5AD1-8C69-E692B643D8B7

#### Ascocotyle

Looss, 1899

D115FB77-D890-58DE-9EC2-CBDD740AFC52

#### Ascocotyle italica

Alessandrini, 1906

FBE75F0F-6CDC-5E12-8028-B98167B70BA4

Parascocotyle italica (Alessandrini, 1906) Price, 1932

##### Parasite of

mammals - Canidae: *Canislupusfamiliaris*.

**Site of infection**: intestine.

##### Distribution

Occurring in Europe, Africa; **in Georgia**: WG: Kolkheti Lowland reported by Burjanadze (1943) and Kurashvili (1984b).

#### Ascocotyle longa

Ransom, 1920

F3293748-B102-52F0-B38B-E0F06C2CF23C

Parascocotyle longa (Ransom, 1920) Stunkard & Haviland, 1924
Metascocotyle witenbergi Ciurea, 1933

##### Parasite of

mammals - Felidae: *Feliscatus*.

fishes - Cyprinidae: *Bliccabjoerkna*, *Luciobarbusmursa*.

**Site of infection**: gills, intestine, muscles.

##### Distribution

Occurring in Europe, Asia, Africa, America; **in Georgia**: EG: Rivers: Alazani, Mtkvari; WG: Kolkheti reported by Gamtsemlidze (1941), Burjanadze (1943), Chiaberashvili (1957), Chiaberashvili (1968) and Murvanidze et al. (2018).

#### Metagonimus

Katsurada, 1912

B3EB3148-1D8E-57D5-B72D-2FA16860ECB4

#### Metagonimus ciureanus

(Witenberg, 1929) Price, 1931

924B8951-1BEA-54DC-9B79-FB8EB22B366B

Dexiogonimus ciureanus Witenberg, 1929

##### Parasite of

mammals - Canidae: *Canisaureus*, *Vulpesvulpes*; Felidae: *Felischaus*.

**Site of infection**: intestine.

##### Distribution

Occurring in Asia; **in Georgia**: EG: Adigeni, Bolnisi, Marneuli, Rivers: Khrami, Mashavera, Mtkvari; Tbilisi – Samgori reported by Rodonaia (1966b), Rodonaia (1971) and Kurashvili (1984b).

#### Metagonimus yokogawai

(Katsurada, 1912) Katsurada, 1912

7D3025AE-A51B-569E-B9C0-2EC402F2D2E2

##### Parasite of

mammals - Canidae: *Canislupusfamiliaris*; Felidae: *Feliscatus*.

molluscs (intramolluscan stage) - Melanopsidae: *Melanopsispraemorsa*.

**Site of infection**: intestine.

##### Distribution

Holarctic distribution; **in Georgia**: WG: Batumi, Kolkheti, Rivers of western Georgia reported by Gamtsemlidze (1941), Burjanadze (1943), Kurashvili (1984b) and Ataev and Dobrovolskiy (1992).

#### Pygidiopsis

Looss, 1907

CF9ADA99-3BEF-5DB3-80E0-89111A2F0F16

#### Pygidiopsis genata

Looss, 1907

F965E9B6-11A2-523E-98F8-69732B50E12C

##### Parasite of

fishes (metacercariae) - Gobiidae: *Babkagymnotrachelus*, *Neogobiusmelanostomus*.

**Site of infection**: gills, muscles.

##### Distribution

Occurring in Europe, Africa; **in Georgia**: WG: Paliastomi Lake reported by Chernova (1973), Kurashvili and Petriashvili (1977) and Murvanidze et al. (2018).

#### Opisthorchiidae

Looss, 1899

4E421E11-26DC-560F-95AE-1910EFA27635

#### Apophallus

Lühe, 1909

B48373EC-C46B-59A4-BFBB-7BB2DAB582B2

#### Apophallus donicus

(Skrjabin & Lindtrop, 1919) Price, 1931

F2AD06D5-3507-5BB9-AF84-0A59B1EE28CC

##### Parasite of

fishes (metacercariae) - freshwater fishes.

**Site of infection**: fins, skin.

##### Distribution

Holarctic distribution; **in Georgia**: WG: freshwaters of West Georgia reported by Chiaberashvili (1955) and Murvanidze et al. (2018).

#### Apophallus muehlingi

(Jägerskiöld, 1899) Lühe, 1909

15B89A3E-6ED6-5AE4-B774-8EE8D986CC8E

##### Parasite of

fishes (metacercariae) - Cyprinidae: *Abramisbrama*.

**Site of infection**: fins, skin.

##### Distribution

Palaearctic distribution; **in Georgia**: WG: Paliastomi Lake reported by Chernova (1977), Kurashvili and Petriashvili (1977) and Murvanidze et al. (2018).

#### Cryptocotyle

Lühe, 1899

27D9483D-9BAA-5858-9383-8D7421DDE5F4

#### Cryptocotyle concava

(Creplin, 1825) Lühe, 1899

E9496916-7CA3-5CB8-888F-64395B06ED8D

##### Parasite of

fishes (metacercariae) - Gobiidae: *Babkagymnotrachelus*, *Neogobiusmelanostomus*.

**Site of infection**: skin, musculature.

##### Distribution

Holarctic distribution; **in Georgia**: WG: Paliastomi Lake reported by Chernova (1977), Kurashvili and Petriashvili (1977) and Murvanidze et al. (2018).

#### Metorchis

Looss, 1899

1B935DF9-883E-5A06-B23D-0F40B9E90A98

#### Metorchis bilis

(Braun, 1790) Odening, 1962

64CC396C-4EAC-5EDF-B602-227DC594B042

##### Parasite of

fishes (metacercariae) - Cyprinidae: *Cyprinuscarpio*, *Rutilusrutilus*, *Scardiniuserythrophthalmus*.

**Site of infection**: muscle.

##### Distribution

Holarctic distribution; **in Georgia**: WG: Didi Narionali Lake, Paliastomi Lake reported by Chernova (1977), Kurashvili and Petriashvili (1977) and Murvanidze et al. (2018).

#### Metorchis xanthosomus

(Creplin, 1846) Braun, 1902

375B1ADA-E84E-5F83-BA5E-7A8AC1477AF9

Metorchis intermedius Heinemann, 1937

##### Parasite of

birds - Anatidae: *Anasacuta*, *A.crecca*, A.plathyrynchosf.domestica, *Anseranser*, *Aythyafuligula*.

**Site of infection**: gallbladder, hepatic ducts.

##### Distribution

Occurring in Europe; **in Georgia**: EG: Marneuli; WG: Abasha – Tskhenistskali, Samtredia, Zugdidi reported by Kurashvili (1957), Kurashvili (1961a), Kurashvili et al. (1976) and Kurashvili (1984b).

#### Opisthorchis

Branchard, 1895

22BFD2AE-5A27-584A-A4BF-A99C72D61D3B

#### Opisthorchis geminus

(Looss, 1896) Looss, 1899

B2CED2A1-2D56-592E-9EE0-8573FB4B2E13

##### Parasite of

birds - Ardeidae: *Ardeollaralloides*.

**Site of infection**: gallbladder, hepatic ducts.

##### Distribution

Occurring in Southeast Asia, Africa; **in Georgia**: EG: Gardabani – Jandari Lake, surroundings of Tbilisi reported by Kurashvili (1957) and Kurashvili (1961a).

#### Opisthorchis simulans

(Looss, 1896) Kowalewski 1898

7E7DA889-6E90-58EE-9CA6-DC3713EB1F45

##### Parasite of

birds - Anatidae: Anasplathyrynchosf.domestica.

**Site of infection**: hepatic ducts.

##### Distribution

Occurring in Africa; **in Georgia**: WG: Samtredia reported by Kurashvili et al. (1976) and Kurashvili (1984b).

#### Paramphistomoidea

Fischoeder, 1901

1497BECF-DDC9-557C-93F3-ED465FE4DC4D

#### Diplodiscidae

Cohn, 1904

5F818966-0CDE-50E8-B5FE-A2960800B6CE

#### Diplodiscus

Diesing, 1836

4CBE1C18-4153-5E81-A72F-05171BE2F5C2

#### Diplodiscus mehrai

Pande, 1937

AE286026-DE17-52F8-9BD0-E53CB7D7ADCA

##### Parasite of

amphibians - Ranidae: *Pelophylaxridibundus*.

**Site of infection**: large intestine, rectum.

##### Distribution

Occurring in Europe and India; **in Georgia**: EG: Aragvi River Basin, Bazaleti Lake, Tbilisi – Samgori reported by Chiaberashvili and Mchedlidze (1961), Petriashvili (1964), Burtikashvili and Getzadze (1981), Petriashvili et al. (1985), Sey (2001) and Murvanidze et al. (2008a).

#### Diplodiscus subclavatus

(Pallas, 1760) Diesing, 1836

CB391EF5-7D99-507F-AC7D-49C879DE08F1

##### Parasite of

reptiles - Colubridae: *Natrixnatrix*.

amphibians - Bufonidae: *Bufotesviridis*; Ranidae: *Pelophylaxridibundus*, *Ranamacrocnemis*.

mollusc (intramolluscan stage) - Planorbidae: *Planorbisplanorbis*.

**Site of infection**: large and small intestine, rectum.

##### Distribution

Occurring in Europe, Asia, Africa; **in Georgia**: EG: Aragvi River Basin, Bazaleti Lake, Borjomi, Jinvali, Kazbegi, Kodjori, Samgori, Sartichala – River Iori, surroundings of Tbilisi; WG: Gali, Khobi, Ozurgeti, Senaki, surroundings of Batumi, Tkibuli Reservoir, Zugdidi reported by Chiaberashvili and Mchedlidze (1961), Petriashvili (1964), Petriashvili (1966), Chiaberashvili (1971a), Burtikashvili and Getzadze (1981), Giorgadze (1985), Jankarashvili (1985), Petriashvili et al. (1985), Sey (2001), Murvanidze et al. (2008a) and Murvanidze et al. (2008b).

#### Paramphistomidae

Fischoeder, 1901

CDA0F3E7-DF96-5249-B2F8-072C171F51B2

#### Calicophoron

Näsmark, 1937

9CB40239-9C7B-5977-85B0-E48AAB15517A

#### Calicophoron calicophorum

(Fischoeder, 1901) Näsmark, 1937

E5845EBF-FF7D-5C4D-9D53-B9ADD4A93640

##### Parasite of

mammals - cattle.

molluscs (intramolluscan stage) - Planorbidae: *Planorbisplanorbis*.

**Site of infection**: omasum, rumen.

##### Distribution

Occurring in Asia, Africa, Oceania; **in Georgia**: EG: Akmeta, Gardabani, Gurjaani, Krtsanisi, Kvareli, Lagodekhi, Signagi, Telavi, Teritskaro, Tsalka; WG: Khobi, Lanchkhuti, Samtredia reported by Kurashvili (1984b), Sey (2001) and Fotskhveria (2002).

#### Paramphistomum

Fischoeder, 1901

C476D24B-5BE8-524A-81B7-2F49AF729902

#### Paramphistomum cervi

(Zeder, 1790) Fischoeder, 1901

8F205EFD-0449-5F88-90C0-9DBFE1A5F8EA

##### Parasite of

mammals - Bovidae: *Bostaurus*, *Oviesaries*; Cervidae: *Capreoluscapreolus*.

molluscs (intramolluscan stage) - Planorbidae: *Planorbisplanorbis*.

**Site of infection**: omasum, rumen, small intestine, stomach.

##### Distribution

Cosmopolitan distribution; **in Georgia**: EG: Bakuriani; WG: Akhalqalaqi, Khobi (Qarieta), Lanchkhuti, Poti, Samtredia, Sokhumi, Qutaisi reported by Gamtsemlidze (1941), Burjanadze (1943), Rodonaia (1962), Rodonaia (1966a), Chiaberashvili (1971a), Kurashvili (1984b) and Sey (2001).

#### Paramphistomum scotiae

Willmott, 1950

97215685-B374-5A93-B2F0-FE9D8C45682C

Liorchis scotiae (Willmott,1950) Velichko, 1966

##### Parasite of

mammals - Cervidae: *Capreoluscapreolus*.

**Site of infection**: rumen.

##### Distribution

Occurring in Europe; **in Georgia**: WG: Khobi, Poti, Qarieta reported by Rodonaia (1971) and Kurashvili (1984b).

#### Paramphistomum skrjabini

Popova, 1927

AF211979-3515-5B36-B720-680A070E4C1C

##### Parasite of

mammals - Bovidae: *Bostaurus*, *Bubalusbubalis*.

molluscs (intramolluscan stage) - Planorbidae: *Planorbisplanorbis*.

**Site of infection**: rumen.

##### Distribution

Reported from Georgia only; **in Georgia**: EG: Eastern Georgia reported by Burjanadze (1943), Rodonaia (1960) and Rodonaia (1971).

#### "fam. Paramphistomidae" gen. sp.

9BA8A378-A99A-5FFB-B08E-80636E3A0921

##### Parasite of

molluscs (intramolluscan stage) - Planorbidae: *Planorbisplanorbis*.

**Site of infection**: no data.

##### Distribution

Cosmopolitan distribution; **in Georgia**: EG: Gori, Khashuri, Lagodekhi, Manglisi, Qareli, Tetritskaro, Tsalka reported by Matsaberidze et al. (1989).

#### Plagiorchioidea

Lühe, 1901

67B59498-36C2-58E4-993A-59D4C68CA6DE

#### Brachycoeliidae

Looss, 1899

D553C4FC-9424-5D50-9294-B94F9ABCEF55

#### Brachycoelium

Dujardin, 1845

2DBF93CC-EAA4-553C-9CA4-174EF5875FEE

#### Brachycoelium salamandrae

(Frölich, 1789) Lühe, 1909

C09563D9-BF4B-5AC8-83C7-E24B14E75823

##### Parasite of

reptiles - Salamandridae: *Mertensiellacaucasica*.

**Site of infection**: small intestine.

##### Distribution

Occurring in Europe, North America, North Africa; **in Georgia**: EG: Bakuriani reported by Petriashvili et al. (1985) and Murvanidze et al. (2008b).

#### Cephalogonimidae

Looss, 1899

F775E447-6698-5EB9-AD7E-7A1EE027E46C

#### Cephalogonimus

Poirier, 1886

323F9979-2BF6-5516-93E5-973EF3AE30B7

#### Cephalogonimus europaeus

Blaizot, 1910

33A96023-8657-54D7-8181-A1A9C8A7374E

##### Parasite of

amphibians - Ranidae: *Pelophylaxridibundus*.

molluscs (intramolluscan stages) - Lymnaeidae: *Galbatruncatula*.

**Site of infection**: gallbladder, hepatopancreas, intestine.

##### Distribution

Occurring in Europe; **in Georgia**: EG: Samgori, Sartichala; WG: Ozurgeti reported by Chiaberashvili and Mchedlidze (1961), Chiaberashvili (1971a) and Kurashvili (1984b).

#### Cephalogonimus retusus

(Dujardin, 1845) Odhner, 1910

D3905400-D6AC-5DE3-830F-7068F146BBED

##### Parasite of

amphibians - Ranidae: *Pelophylaxridibundus*.

**Site of infection**: small intestine.

##### Distribution

Occurring in Europe; **in Georgia**: EG: Aragvi River Basin, Bazaleti Lake, Tbilisi – Samgori; WG: Samtredia, Tkibuli Reservoir reported by Chiaberashvili and Mchedlidze (1961), Petriashvili (1964), Burtikashvili and Getzadze (1981), Kurashvili (1984b), Giorgadze (1985), Petriashvili et al. (1985) and Murvanidze et al. (2008a).

#### Cephalogonimus spp.

A5357825-34A7-59B9-8D8F-96180CCC861C

##### Parasite of

amphibians - Ranidae: *Pelophylaxridibundus*.

mollusc (intramolluscan stage) - Lymnaeidae: *Lymnaeastagnalis*.

**Site of infection**: body cavity, liver, lymph sac, small intestine.

##### Distribution

Ocurring in North and South America, India; **in Georgia**: EG: River Iori, Sartichala, Tabatskuri Lake reported by Javelidze and Chiaberashvili (1985) and Murvanidze et al. (2008a).

#### Haematoloechidae

Freitas & Lent, 1939

DF4510E8-0811-5A62-8D57-9AF2A33595E1

#### Haematoloechus

Looss, 1899

DA206966-AE63-51A0-B0B5-16690EC8F508

Pneumonoeces Looss, 1902

#### Haematoloechus asper

Looss, 1899

1EADBCB6-F52B-519C-A64B-F348DD05376C

##### Parasite of

amphibians - Bufonidae: *Bufobufo*; Ranidae: *Pelophylaxridibundus*.

**Site of infection**: lungs.

##### Distribution

Occurring in Europe; **in Georgia**: EG: Aragvi River Basin, Jandari Lake reported by Burtikashvili et al. (1978), Burtikashvili and Getzadze (1981), Petriashvili et al. (1985) and Murvanidze et al. (2008a).

#### Haematoloechus variegatus

(Rudolphi, 1819) Looss, 1899

8E897449-83C7-5A2D-A4BA-8F74C5952567

##### Parasite of

amphibians - Bufonidae: *Bufobufo*, *Bufotesviridis*; Ranidae: *Pelophylaxridibundus*, *Ranamacrocnemis*.

**Site of infection**: lungs.

##### Distribution

Occurring in Europe, Asia; **in Georgia**: EG: Aragvi River Basin, Lakes: Bazaleti, Jandari; Kumisi Reservoir, Samgori, surroundings of Tbilisi; WG: Ozurgeti, Poti, Samtredia, Tkibuli Reservoir reported by Chiaberashvili and Mchedlidze (1961), Petriashvili (1964), Kurashvili et al. (1973), Burtikashvili et al. (1978), Giorgadze (1985), Petriashvili et al. (1985), Kurashvili et al. (1991) and Murvanidze et al. (2008a).

#### Haematoloechus sp.

991AE875-F7CF-55CE-8A8E-6A47D84ACE6C

##### Parasite of

amphibians - Ranidae: *Pelophylaxridibundus*.

**Site of infection**: lungs.

##### Distribution

Cosmopolitan distribution; **in Georgia**: WG: Ozurgeti reported by Kurashvili (1984b).

#### Leptophallidae

Dayal, 1938

0ED69338-07D3-5DEE-8043-9F30D38BEE32

#### Leptophallus

Lühe, 1909

FDE2B6FE-A72A-53C9-B04B-F8627EADDE37

#### Leptophallus nigrovenosus

(Bellingham, 1844) Lühe, 1909

842F3B05-AE7B-56C4-927D-7737A79EFCE0

##### Parasite of

reptiles - Colubridae: *Natrixnatrix*.

**Site of infection**: intestine.

##### Distribution

Palaearctic distribution; **in Georgia**: EG: Jinvali, Kazbegi, Lagodekhi, Surroundings of Tbilisi; WG: Khobi, surroundins of Batumi – Kakhaberi Lake, Zugdidi reported by Sharpilo (1962) and Jankarashvili (1985).

#### Macrodera

Looss, 1899

1B3EA8E6-CDF6-5779-9E03-01AE4DC1D419

#### Macrodera longicollis

(Abildgaard, 1788) Looss, 1899

0EE968AB-73C5-5111-BAFA-5403C3A0C061

##### Parasite of

reptiles - Colubridae: *Natrixnatrix*, *N.tesselata*.

**Site of infection**: lungs, respiratory tract.

##### Distribution

Palaearctic distribution; **in Georgia**: EG: Bazaleti Lake, Jinvali, Kazbegi surroundings of Borjomi, surroundings of Tbilisi; WG: Khobi, Surroundings of Batumi, Zugdidi reported by Sharpilo (1962), Petriashvili (1966), Kurashvili (1984b), Jankarashvili (1985) and Kurashvili et al. (1991).

#### Paralepoderma

Dollfus, 1950

48332B49-2B6B-510A-BA11-56768508C791

#### Paralepoderma cloacicola

(Lühe, 1909) Dollfus, 1950

29481D48-79DE-55BA-A989-C76DF6E725B5

##### Parasite of

reptiles - Colubridae: *Natrixnatrix*, *N.tesselata*.

**Site of infection**: intestine.

##### Distribution

Occurring in the Palaearctic, Africa; **in Georgia**: EG: Borjomi, Jinvali, Kazbegi, surroundings of Tbilisi; WG: Khobi, Kulevi, surroundings of Batumi, Zugdidi reported by Sharpilo (1962), Kurashvili (1984b), Jankarashvili (1985) and Kurashvili et al. (1991).

#### Orientocreadiidae

Yamaguti, 1958

0D5BC93D-AA4C-5AB2-8B95-402A8DE67C54

#### Orientocreadium

Tubangui, 1931

FDDA53D8-96A8-599D-A2A9-E7C3AB6A808A

#### Orientocreadium pseudobagri

Yamaguti, 1934

FC02724E-AD3B-5FCC-AEB1-050A2D6DC7ED

Paratormopsolus siluri Dubinina & Bychowsky, 1954
Orientocreadium siluri (Dubinina & Bychowsky in Skrjabin, 1954) Yamaguti, 1958

##### Parasite of

fishes - Siluridae: *Silurusglanis*.

**Site of infection**: intestine.

##### Distribution

Known from Asia (Iraq); **in Georgia**: WG: Bebesiri Lake, River Tekhura reported by Chiaberashvili (1962b) and Murvanidze et al. (2018).

#### Plagiorchiidae

Lühe, 1901

6AEBC24F-D160-5119-9C4C-0F31C8260090

#### Haplometra

Looss, 1899

AD498D3E-FEA3-516B-B002-63F0ED956F22

#### Haplometra brevicaeca

Timon-David, 1962

6A8E01F2-CF77-576D-B3E0-34CE5F437722

##### Parasite of

amphibians - Ranidae: *Ranamacrocnemis*.

**Site of infection**: lungs.

##### Distribution

Occurring in Europe; **in Georgia**: EG: Kazbegi, Cross Pass, Minor Caucasus reported by Petriashvili et al. (1985) and Murvanidze et al. (2008a).

#### Plagiorchis

Lühe, 1899

5723E121-1DEB-5E13-8C8A-3AE90E37D33D

#### Plagiorchis maculosus

(Rudolphi, 1802) Braun, 1901

0C7E67B2-41DF-5653-9E11-E83D61B8B7AD

##### Parasite of

birds - Caprimulgidae: *Caprimulguseuropaeusmeridionalis*; Hirundinidae: *Hirundorusticarustica*.

**Site of infection**: intestine.

##### Distribution

Occurrence in the Holarctic Region, India, Australia; **in Georgia**: EG: Gardabani, Lagodekhi National Park, Tbilisi; WG: Samtredia, Surroundings Poti reported by Kurashvili (1957) and Kurashvili (1961a).

#### Plagiorchis vespertilionis

(Müller, 1780) Braun, 1900

AEA5F1E0-85D9-553F-B1E1-6C0B8BB31571

##### Parasite of

mammals - Vespertilionidae: *Eptesicusserotinus*, *Myotismystacinus*, *Nyctalusleisleri*, *N.noctula*, Rhinolophidae: *Rhinolophushipposideros*, *R.mehelyi*, *Vespertiliosubtilis*.

**Site of infection**: small intestine.

##### Distribution

Holarctic distribution; **in Georgia**: EG: Akhmeta, Bolnisi, Gomareti, Lagodekhi reported by Matsaberidze (1961), Rodonaia (1966b), Matsaberidze and Khotenovskii (1967) and Matsabaridze (1976).

#### Plagiorchis sp.

F4842AF2-EE97-56EA-9BC3-DE3B35C602D0

##### Parasite of

molluscs (intramolluscan stage) - Lymnaeidae: *Radixeuphratica*.

**Site of infection**: body cavity, liver.

##### Distribution

Cosmopolitan distribution; **in Georgia**: EG: Turtle Lake reported by Arabuli, unpublished data 2021.

#### Skrjabinoeces

Sudarikov, 1950

E73853B9-71B2-550A-BDC8-A717274F3292

#### Skrjabinoeces similis

Looss, 1899

5B5867A4-31F7-594E-8FE7-6D02FECBF92E

##### Parasite of

molluscs (intramolluscan stage) - Planorbidae: *Planorbisplanorbis*.

**Site of infection**: no data.

##### Distribution

Palaearctic distribution; **in Georgia: EG**: Rustavi reported by Tkachenko (1990).

#### Telorchiidae

Looss, 1899

AED0DB82-46C5-5C20-829F-2DEEADE4E1F3

#### Opisthioglyphe

Looss, 1899

6F57DE51-46DB-5EF6-B4A1-28906F8856E6

#### Opisthioglyphe ranae

(Frölich, 1791) Looss, 1907

9D4DBBFD-4E9D-5753-8F3F-E7B2B66ABC1F

##### Parasite of

reptiles - Colubridae: *Natrixnatrix*.

amphibians - Bufonidae: *Bufobufo*, *Bufotesviridis*; Hylidae: *Hylaarborea*; Ranidae: *Pelophylaxridibundus*, *Ranamacrocnemis*.

molluscs (intramolluscan stage) - Lymnaeidae: *Galbapalustris*, *Peregrianaperegra*.

**Site of infection**: gall bladder, large intestine, small intestine.

##### Distribution

Occurring in Europe, Asia (Iraq), North Africa; **in Georgia**: EG: Aragvi River Basin, Borjomi, Jinvali, Kazbegi, Kodjori, Samgori, Surroundings of Tbilisi, Tbilisi – Botanic Garden; WG: Batumi, Bebesiri Lake, Gali, Khobi, Lanchkhuti, Ozurgeti, Samtredia, Senaki, Tkibuli Reservoir, Zugdidi reported by Chiaberashvili and Mchedlidze (1961), Javelidze (1964), Burtikashvili and Getzadze (1981), Kurashvili (1984a), Kurashvili (1984b), Giorgadze (1985), Jankarashvili (1985), Petriashvili et al. (1985), Kurashvili et al. (1991) and Murvanidze et al. (2008a).

#### Opisthioglyphe rastellus

(Olsson, 1876) Looss, 1907

CE6E10F1-4B0C-5C67-9C1C-2DDE4C9DD747

Dolichosaccus rastellus (Olsson, 1876) Travassos, 1930

##### Parasite of

amphibians - Ranidae: *Pelophylaxridibundus*, *Ranamacrocnemis*.

molluscs (intramolluscan stage) - Lymnaeidae: *Ampullaceanabalthica*.

**Site of infection**: intestine, hepatopancreas.

##### Distribution

With Palaearctic distribution; **in Georgia**: EG: Central Caucasus (Georgia); Samgori reported by Chiaberashvili (1971a) and Petriashvili et al. (1985).

#### Telorchis

Lühe, 1899

37221895-4033-597D-BA23-B191573F3697

#### Telorchis assula

(Dujardin, 1845) Dollfus, 1957

05B90834-16CA-54DB-891D-1516AC38D3C5

##### Parasite of

reptiles - Colubridae: *Natrixnatrix*, *N.tesselata*; Emydidae: *Emysorbicularis*.

**Site of infection**: intestine.

##### Distribution

Palaearctic distribution; **in Georgia**: EG: Borjomi, Jinvali, Kazbegi, Lagodekhi, Tbilisi, Lakes: Bazaleti, Jandari; WG: Anaklia, Khobi, Kulevi, surroundings of Batumi, Zugdidi reported by Sharpilo (1962), Petriashvili (1966), Kurashvili et al. (1975), Kurashvili (1984b), Jankarashvili (1985), Kurashvili et al. (1991) and Murvanidze et al. (2008b).

#### Telorchis stossichi

Goldberger, 1911

27ADCD30-67C1-5524-8D02-20CE570E7901

##### Parasite of

reptiles - Emydidae: *Emysorbicularis*.

**Site of infection**: intestine.

##### Distribution

Occurring in Europe; **in Georgia: EG**: Lakes: Bazaleti, Jandari reported by Petriashvili (1966), Kurashvili et al. (1975), Jankarashvili (1978) and Murvanidze et al. (2008b).

#### Telorchis spp.

5E4B1A09-2308-5DDF-A509-77727E8E93A0

##### Parasite of

reptiles - Lacertidae: *Lacertastrigata*.

molluscs (intramolluscan stage) - Lymnaeidae: *Radixeuphratica*.

**Site of infection**: intestine, liver.

##### Distribution

In North and South America, Europe, Asia; **in Georgia**: EG: Lakes: Jandari, Turtle reported by Kurashvili et al. (1975) and Arabuli, unpublished data 2021.

#### Pronocephaloidea

Looss, 1899

B1227213-AB53-5F67-99C7-AD051A81516A

#### Notocotylidae

Lühe, 1909

03EB6515-55B6-5A64-A955-520B07FAD9A5

#### Notocotylus

Diesing, 1839

52023AD8-4DF1-5761-98BC-6A9D0E65DB94

#### Notocotylus attenuatus

(Rudolphi, 1809) Kossack, 1911

8134727E-9B06-5110-A451-433C3DAB85DA

##### Parasite of

birds - Anatidae: *Anasclypeata*, *A.plathyrynchos*, A.plathyrynchosf.domestica, *Anseranser*, *Aythyafuligula*, *A.ferina*, *Cygnuscygnus*.

molluscs (intramolluscan stage) - Lymnaeidae: *Lymnaeastagnalis*, *Peregrianaperegra*, *Radixauricularia*.

**Site of infection**: caecum, large intestine, rectum (birds), hepatopancreas (molluscs).

##### Distribution

Cosmopolitan distribution; **in Georgia**: EG: Bolnisi, Dedoplistskaro – Qvemo qedi, Dmanisi, Gardabani, Marneuli, Tetritskaro, Tsalka – Khrami Reservoir, Lakes: Bazaleti, Paravani; WG: Lakes: Bebesiri, Madatafa; Samtredia, surroundings of Poti – Paliastomi Lake reported by Burjanadze (1943), Kurashvili (1957), Kurashvili (1961a), Japaridze and Savateeva (1967), Kurashvili et al. (1976), Kurashvili (1984a), Kurashvili (1984b), Tkachenko (1988) and Arabuli et al. (2015).

#### Notocotylus ephemera

(Nitzsch, 1817) Harwood, 1939

D76B80E2-7709-56BC-88C7-F15B97980BA7

##### Parasite of

molluscs (intramolluscan stage) - Planorbidae: *Planorbisplanorbis*.

**Site of infection**: no data.

##### Distribution

Occurring in Europe; **in Georgia**: EG: Gori – Khidistavi reported by Chiaberashvili (1971a).

#### Notocotylus noyeri

Joyeux, 1922

37B160AF-2A15-59EE-826A-289B7BD21B30

##### Parasite of

mammals - Cricetidae: *Arvicolaterrestris*.

**Site of infection**: small intestine.

##### Distribution

Occurrence in Europe; **in Georgia**: EG: Gori – Khidistavi reported by Matsabaridze (1976).

#### Notocotylus ponticus

Tshiaberashwili, 1964

A6BFD6E6-5258-5588-9574-41D65DC67C4C

##### Parasite of

molluscs (intramolluscan stage) - Bithyniidae: *Bithyniatentaculata*.

**Site of infection**: no data.

##### Distribution

Recorded only in Georgia; **in Georgia**: WG: Bebesiri Lake reported by Chiaberashvili (1971b), Kurashvili (1984a) and Cribb (1991).

#### Notocotylus zduni

Chiaberashvili & Dzavelidze, 1968

CE5635EE-32B5-57D4-A522-B9A0463E1F8B

##### Parasite of

molluscs (intramolluscan stage) - Neritidae: *Theodoxusfluviatilis*.

**Site of infection**: no data.

##### Distribution

Occurrence in Europe; **in Georgia**: WG: West Georgia reported by Chiaberashvili and Javelidze (1968).

## Analysis

### Results

Based on data from literature and our new records, digeneans in Georgia are currently represented by 186 taxa, of which 173 are identified to species level, belonging to 108 genera, 47 families and 17 superfamilies. The majority of trematode species comprised adult stages (160 species), a small proportion being made up by cercariae (33) or metacercariae (24) in their first and second intermediate hosts, respectively. For 28 species (15%), at least two life-cycle stages were recorded (adult in combination with cercariae/metacercariae). Birds and mammals were recorded as definitive hosts only, while the other groups were recorded as definitive and also as second intermediate hosts - fish hosted 32 species as adults and 18 as metacercariae, amphibians hosted 23 species as adults and two as metacercariae and reptiles hosted nine species as adults and three as metacercariae. A total of 35 trematode species were recorded in molluscs, which were used as first or second intermediate hosts. Predominantly, freshwater digeneans were recorded (154 species), while a much lower number of marine (12) and terrestrial (21 species) digeneans was found. A total of 202 free-living vertebrate and mollusc species were recorded as hosts for digeneans – fish of 57 species, amphibians of six species, reptiles of four species, birds of 74 species, mammals of 40 species and molluscs of 21 species.

## Discussion

In the present study, we compiled data on digenean trematode fauna of Georgia, which is represented by 186 species. Amongst these, 37 species belong to the order Diplostomida and 149 species to Plagiorchiida. Predominantly, trematode species using birds as hosts were recorded (62 species), followed by those parasitising fishes (50), mammals (33) and amphibians (25) and the fewest species were reported from reptiles (12), a trend common in trematodes (see Yamaguti (1971)). Adult digeneans, recorded together with a metacercarial and/or cercarial stage, comprised 28 species (15%), which indicates that the Georgian fauna of trematodes and their life-cycles are understudied and large-scale investigations of molluscs are needed to assess the trematode species spectrum completing their life-cycles in Georgia. The majority of species were recorded as associated with freshwater, most probably because of lack of investigation in the marine realm.

Apart from well-identified species, there are taxa of dubious identity appearing in the Georgian scientific literature, which we decided not to include in the checklist above. One of them is *Clinostomum* sp. of Kurashvili (Kurashvili 1950, Kurashvili 1953a, Kurashvili 1957, Kurashvili 1961a), which was not identified to species level, but it was claimed to be closest to *Clinostomumhornum*; however, there is no voucher specimen available for checking, leaving the question of the identity of this record unanswered. The next record is *Petasigerjubilarum* (Elperina in Skrjabin, Petrov & Baschkirova, 1947) Skrjabin & Baschkirova, 1956 found in *Anasplathyrynchos* from Paliastomi Lake (Kurashvili 1957, Kurashvili 1961a, Kurashvili 1984b); however, this species is currently considered a species inquirendum (see Faltýnková et al. (2008)). In another study, Javelidze (1976) obtained an adult experimentally from *Melanopsispraemorsa* and presumed it could be *Philophtalmus* sp. (*nyrocae*? Yamaguti, 1934); however, there is no clear species delimitation.

A specific topic in the literature on digeneans are reports of cercariae (the motile larval stages of digenean trematodes emerging from mollusc hosts). Due to the historical development of understanding of trematode life-cycles, the nomenclature and taxonomy of cercariae was originally separated from that of adult trematodes. This resulted in a substantial amount of cercarial names, many of which now have to be regarded as provisional, unless they can be assigned to a valid species described, based on an adult. In Georgia, surveys of larval trematodes from snails were carried out and some of the cercariae found can be identified to genus level or only to family: *Cercariamonostomi* Linstow, 1884 *ex* *Ampullaceanabalthica* and *A.lagotis* from surroundings of Sartichala (Chiaberashvili 1971a) belongs to the Notocotylidae; *Cercariastylosa* Linstow, 1884 (Chiaberashvili 1971a) could possibly belong to *Ichtyocotylurus* sp.; while *Cercarialimnaeaeovatae* Linstow, 1884 *ex* *A.balthica* (Chiaberashvili 1971a) most probably belongs to Plagiorchiidae (for all compare with Cichy et al. (2011). The following cercariae most probably belong to the family Lecithodendriidae: *Cercaria ksaniensis ex Melanopsispraemorsa* from the River Qsani (Eastern Georgia) (Javelidze 1973), *Cercariathezamiensis* from River Thezami (Eastern Georgia) (Javelidze 1973), *Cercariarosetae* and *Cercaria ginetzinskaiae ex M.praemorsa* from the River Qsani (Javelidze and Chiaberashvili 1973) and *Cercariacolchica* I *ex* *Peregrianaperegra* from Kolkheti lowland water reservoirs (Kurashvili 1984a). There were more cercariae recorded which could not be assigned to any genus or family and, because of lack of information, we do not make any attempt for grouping into families; they are the following: *Cercaria samgoriensis ex A.balthica* from surroundings of Samgori (Chiaberashvili 1971a); *Cercaria joriensis ex A.balthica* and *P.planorbis* from Sartichala and Gardabani (Chiaberashvili 1971a); *Cercaria burti ex P.planorbis* from Samgori (Chiaberashvili 1971a); *Cercariagracilis ex A.balthica* from surroundings of Sartichala and Gardabani (Chiaberashvili 1971a); *Cercariarhionica* I from River Pichori (Kurashvili 1984a); *Cercariarhionica* VIII Olenov & Doborovolskiy, 1975 and *Cercariarhionica XII* Olenov & Doborovolskiy, 1975 *ex* *M.praemorsa* from rivers of Western Georgia (Ataev and Dobrovolskiy 1992); *Cercariaascanica* Chiaberashvili & Javelidze 1977 *ex* *M.praemorsa* (Chiaberashvili and Javelidze 1977). Many of them were designated by the authors (Chiaberashvili 1971a, Javelidze 1973, Javelidze and Chiaberashvili 1973, Kurashvili 1984a) as new species, however, without providing any adults as vouchers.

Our revision of digenean species listed in the monograph by Kurashvili et al. (1980) revealed that some of the data on species distribution are vague and inaccurate. This is mainly because the transboundary River Mtkvari (Kura) and the mountain ranges (Caucasus Mountains), which stretch over all Caucasian countries, are frequently considered a continuous distributional area for the taxa and their locations were not distinguished by country. As a result, subsequent sources, citing Kurashvili et al. (1980) and listing the Caucasian countries for species distribution, are frequently imprecise. For instance, according to the NHM London database (NHML 2022), all digenean species recorded in the South Caucasus, based on Kurashvili et al. (1980), are also indicated for Georgia. However, after checking the original literary sources (Kurashvili et al. 1951, Chiaberashvili 1955, Chiaberashvili 1957, Chiaberashvili 1959, Qoiava 1966, Chiaberashvili 1968, Petriashvili 1971), we found out that the following digenean species (that were actually recorded in Azerbaijan) were not reported from Georgia: *Acanthocreadiumaraxicus* Mikailov, 1969; *A.talischiensis* Mikailov, 1969; *Ascocotylecoleostoma* (Looss, 1896) Looss, 1899; *Asymphylodoraimitans* (Muhling, 1898) Looss, 1899; *A.kurensis* Paschajev, 1970; *Bunocotylecingulata* Odhner, 1928; *Bychowskycreadiumbychowsky* Mikailov, 1968; *B.schiliani* Mikailov, 1967; *Hysteromorphatriloba* (Rudolphi, 1819) Lutz, 1931; *Ichthyocotyluruspileatus* (Rudolphi, 1802) Odening, 1969 and *Sanguinicolainermis* Plehn, 1905. Similar results were found for host species and their distribution. Careful checking of the original literature (Petriashvili 1964, Petriashvili 1966) cited by Sey (2001) revealed that *R.esculenta* is not a host for *D.mehrai* in Georgia; while *Emysorbicularis* and *Triturusvulgaris* are not hosts of *D.subclavatus*; however, they are listed as such in the database of NHM London. This highlights the continuing importance of assessing primary sources.

In recent years, we recorded trematode intramolluscan stages (sporocysts, rediae, cercariae and metacercariae) in snails, which were new host records for trematodes in Georgia: *Helixlucorum* for Brachylaimidae (Murvanidze et al. 2010, Arabuli et al. 2019); *Oxychilusmingrelicus* for *Dicrocoelium* sp. (Arabuli 2018); *Lymnaeastagnalis* for *Diplostomumspathaceum*, *Moliniellaanceps* and *Notocotylusattenuatus*; *Radixeuphratica* for *Telorchis* sp., *Tylodelphys* sp. and *Plagiorchis* sp. (Arabuli, unpublished data). *Moliniellaanceps* (Molin, 1859) Hübner, 1939 was recorded for the first time in Georgia by Arabuli et al. (2015).

**Conclusion**

Data on the digeneans of Georgia are mainly represented by adults in their definitive hosts and they are predominantly from freshwater. Most of the research was conducted many decades ago and was based solely on morphology and was published predominantly in Georgian or Russian language. Since there is a striking shortage of studies on intermediate hosts (i.e. molluscs), large-scale and site-intensive investigations are needed to explore species diversity and distribution and to obtain data on life-cycles and transmission pathways to be further used for ecological and epidemiological studies, as well as for biodiversity conservation in Georgia.
